# CHIP-seq and transcriptomics reveal a new role of circadian-regulated StBBX24 protein in potato reproduction

**DOI:** 10.1186/s12870-025-07811-0

**Published:** 2025-12-02

**Authors:** Klaudia Grądzka, Magdalena Biegańska, Grzegorz Koczyk, Agata Młodzińska, Izabela Pawłowicz, Agnieszka Kiełbowicz-Matuk

**Affiliations:** 1 Institute of Plant Genetics, Polish Academy of Sciences, Strzeszyńska 34, 60-479, Poznań, Poland; 2 Bioidea, Warszawa, 02-991 Poland

**Keywords:** B-box protein, CHIP-seq, Flowering time, Tuber development, RNA-seq, *Solanum tuberosum*

## Abstract

**Background:**

The flowering and tuberization are controlled by common determinants that mediate the regulation of both processes. Among transcription regulators involved in light-dependent aspects of growth and development are B-box zinc finger proteins. To gain a better understanding of the StBBX24 role in flowering and tuber formation, we aimed to investigate the regulatory network of StBBX24 in apical shoot parts at different stages of development and in stolons at the hook stage of tuber formation in *StBBX24*-silenced and -overexpressed lines using RNA-seq. In addition, we intended to identify StBBX24-targeted genes specific to the light-dark cycle and potentially involved in potato reproduction by performing CHIP-seq.

**Results:**

The genome-wide analysis of StBBX24 binding sites identified numerous light- and dark-specific targets, among them those participating in the induction of flowering and floral development. RNA-seq analysis of the apical shoot parts of *StBBX24*-silenced and -overexpressed lines revealed substantial modifications in the expression of genes functioning in the flowering process. Furthermore, hook-stage stolons transcriptomics of the transgenic lines revealed that the StBBX24 protein also affects the tuberization process through alteration of the expression of genes participating in stolon-to-tuber transition.

**Conclusions:**

Altogether, these data reveal that StBBX24 is involved in potato flowering repression and impedes the formation of tubers. Our results provided a more comprehensive understanding of the molecular basis of potato reproduction and the participation of the StBBX24 protein in this process.

**Supplementary Information:**

The online version contains supplementary material available at 10.1186/s12870-025-07811-0.

## Background

Light is the predominant environmental factor that regulates plant growth and development. Plants sense and adapt to the diurnal periodicity of light intensity and variations in day length. The circadian-dependent transition from the vegetative to the generative phase is critical to plant persistence [1].

There is strong evidence, that in potato (*Solanum tuberosum*) flowering and tuberization are under the control of common determinants that mediate the regulation of both processes [2–4]. Those processes are initiated by the synthesis of mobile signal molecules, florigen and tuberigen, accumulated in the leaves and then transported to the shoot apical meristem (SAM). The major components of this mobile signal are the FT (FLOWERING LOCUS T) proteins. Till now, six *FT-like* genes, called *SELF PRUNING* (*SP*), have been identified in the potato genome, including *StSP6A*,* StSP3D/SFT*,* StSP5G* (*StSP5G-A*), *StSP5G-B*, *StSP5G-like* and *StFTL1* (*FLOWERING LOCUS T-Like 1*) [5–8]. They are members of the phosphatidylethanolamine-binding protein (PEBP) family and play a significant role in flowering induction and storage organ formation [6, 9]. As shown, *StSP6A* encodes a protein that acts as a tuberigen signal promoting tuber formation [7], while *StSP3D* encodes a protein that acts as a florigen signal promoting floral development [4]– [5]. Recently, Jing et al. [8] uncovered the function of StSP3D and StFTL1 proteins as new photoperiod-dependent long-range tuber-inducing signals evidencing the existence of an alternative to StSP6A signaling pathways [8, 10].

The transcriptional and post-translational regulators regulating *FT* genes expression differ significantly in response to environmental and endogenous stimuli among species [4]– [5, 11]. A major role in this day-length dependent regulation is played by transcriptional repressors belonging to various families of TFs (TRANSCRIPTION FACTORs), including MADS domain transcription factors, i.e., SVP (SHORT VEGETATIVE PHASE), DOF proteins (DNA-binding with One Finger), i.e., CDF1/CDF2 (CYCLING DOF FACTORS 1/2), TCP (TEOSINTE BRANCHED/CYCLOIDEA/PCF) protein, i.e., StAST1 and B-box domain TFs [4, 12–14]. However, regardless of the considerable difference in developmental changes between taxa, the principal players coordinating developmental timing appear to be conserved. The CO-FT molecular module is the major photoperiodic regulon controlling plant reproduction.

B-box domain proteins (BBXs) form one of the most abundant groups of TFs and regulators that play pivotal roles in light-dependent aspects of plant growth and development directly controlling the transcription of target genes or interacting with other partners to form complex regulatory networks [15–18]. They are involved in seedling photomorphogenesis [19–23], photoperiodic regulation of flowering and tuberization [7, 15, 24]– [25], shade avoidance [26] and various phytohormone-mediated pathways, such as abscisic acid, gibberellins, brassinosteroids, ethylene and jasmonic acid [27]– [28]. In potato, the pivotal role of StBBX1 (also known as StCOL1/StCO/CONSTANS), a B-box type transcription factor, in flowering and tuberization has been reported [7, 15]. Signal derived from light is integrated into the circadian clock and then guided by a photoperiodic pathway to trigger *StBBX1* expression, which in turn controls *StSP6A* gene transcription through direct activation of its repressor, StSP5G protein [7]. Besides the BBX1 (CO), other BBX proteins can also affect flowering time in various plant species. As shown in Arabidopsis, AtBBX4 (COL3), AtBBX7 (COL9), AtBBX10 (COL12), and AtBBX17 (COL8) suppress flowering negatively regulating *CO* expression under long-day [29–32], while AtBBX6 (COL5) induces flowering by increasing *CO* expression under short-day conditions [33]. In rice, OsBBX5 (COL4), OsBBX7 (COL5), OsBBX10 (COL10) and OsBBX23 (COL13) proteins delay flowering by reducing the expression of *FT-*like genes and subsequently impacting heading date by affecting the *Early heading date 1* (*Ehd1*) gene under short-day [34–37]. Furthermore, CmBBX8 protein stimulates flowering in summer-flowering chrysanthemum under long-day conditions, while CmBBX13 and CmBBX24 cause late flowering under long and/or short-day conditions analogous to StBBX24 protein in cultivated potato [38–41].

We previously proposed the biological function of StBBX24 (currently named StBBX20) in cultivated potato (*Solanum tuberosum*, cv. Desirée) as a negative transcriptional regulator of critical genes associated with the floral transition process. We observed earlier flowering of *StBBX24*-silenced lines compared to the wild-type plants. Contrary, lines with *StBBX24* overexpression either did not produce flower buds or revealed late flowering [41]. Moreover, we noticed the differences in the number and size of tubers in both transgenic lines as compared with wild-type plants [41].

To gain a better understanding of the StBBX24 role in flowering and tuber formation, we aimed to investigate the regulatory network of StBBX24 in apical shoot parts at different stages of development and in stolons at the initial stage of tuber formation, known as hook stage, in *StBBX24*-silenced and -overexpressed lines using RNA sequencing (RNA-seq). Moreover, based on our earlier results showing that *StBBX24* expression is subject to diurnal regulation, with a substantial rise in transcript and protein abundance after 8 h of light and a notable decrease after 4 h in darkness, we intended to identify StBBX24-targeted genes specific to the light-dark cycle and potentially involved in potato reproduction by performing chromatin immunoprecipitation coupled with high-throughput sequencing (CHIP-seq). The genome-wide determination of StBBX24 binding sites and global gene expression analysis revealed that the StBBX24 protein impacts flowering by adjusting the expression of genes participating in this process and regulating flower development. Furthermore, we found that the StBBX24 also affects the tuberization process through substantial modifications of the expression of genes like, *StBEL5* (*BEL1-LIKE TRANSCRIPTION FACTOR 5*), *StPOTH1* (*KNOTTED1-TYPE TRANSCRIPTION FACTOR POTATO HOMEOBOX1*), *StGA2ox1* (*Gibberellin 2-oxidase 1*) and *StSP6A*. Thus, our study demonstrated a previously unidentified role of the StBBX24 protein in flowering repression and tuber development, and provides new insights into potato reproduction.

## Methods

### Plant material and growth conditions

*Solanum tuberosum* L., cv. Desirée wild-type (WT) plants and transgenic lines (*StBBX24*-mGFP-His, amiRStBBX24.2.34 and *StBBX24*-OE3) were propagated *in vitro* on solid MS medium at 20/18°C (day/night) under a 150 µmol photons m^−2^ s^−1^ PFD and a 14-hour photoperiod for three weeks. Then, four single-eye plugs from tubers were transferred to the soil (pots diameter 21 cm) and grown in the phytotron under conditions as follows: 20/18°C (day/night), 40% relative humidity, PFD of 350 µmol photons m^−2^ s^−1^ and 14-hour-light/10-hour-dark regime. The term Zeitgeber time (ZT) relates to the experimental time, where the ZT0 point corresponded to light-on (initiation of experimental dawn) and the ZT14 point to light-off (initiation of experimental dusk) [42]. Two independent experiments were conducted and two independent plant samples from each experiment were collected.

For the CHIP-seq assay, well-expanded leaves of a three-week-old transgenic line carrying *StBBX24* full-length coding sequence and double fusion C-terminal tags: mGFP and 6xHis (*StBBX24*-mGFP-His) were collected at ZT6 (6 h of light) and ZT23 (9 h of dark). For the RNA-seq experiment, 5- and 7-week-old apical shoot parts and 4-week-old stolons of WT plants, and two transgenic lines: *StBBX24* gene-silenced line (amiRStBBX24.2.34) and *StBBX24* gene-overexpressed line (*StBBX24*-OE3) were collected at ZT6.

### Vector preparation for transgenic lines generation

To generate transgenic *StBBX24* gene-silenced lines, artificial microRNAs (amiRNAs) targeting the *StBBX24* were designed using Web MicroRNA Designer (www.wmd3.weigelworld.org). *StBBX24* mRNA sequence fragments revealing no allelic polymorphism were chosen and analyzed to find the most effective target sites for RNA-induced silencing complex (RISC) slicing [41]. Using the same platform, three amiRNAs were selected. Then, several overlapping PCRs were carried out using the suitable primers for the three constructs to exchange miR319b/miR319b* for amiRStBBX24.1/amiRStBBX24.1*, amiRStBBX24.2/amiRStBBX24.2*, and amiRStBBX24.3/amiRStBBX24.3* [41]. The DNA fragments carrying amiRStBBX24.1, amiRStBBX24.2, and amiRStBBX24.3 were cloned into the Hannibal vector [43], and then transferred into a pART27 vector along with the CaMV35S promoter and the OCS terminator [44]. Separate transgenic lines containing the T-DNAs with the DNA fragments carrying *amiRStBBX24.1*, *amiRStBBX24.2* or *amiRStBBX24.3* genes were generated and then tested for silencing effectiveness using RT-qPCR (Figure S1 A). Lines showing significantly reduced *StBBX24* transcript level compared to WT plants were selected to analyze the StBBX24 protein presence using Western blot method (Figure S1 B).

The *StBBX24* gene-overexpressed lines (*StBBX24*-OE) were generated in our previous studies by recombining the *StBBX24* full-length coding sequence from the pENTR clone with the pGWB402, Gateway-compatible binary vector [41]. pGWB402 was a gift from Tsuyoshi Nakagawa (Addgene plasmid #74796, http://n2t.net/addgene:74796; RRID: Addgene_74796) [45].

**Fig. 1:**
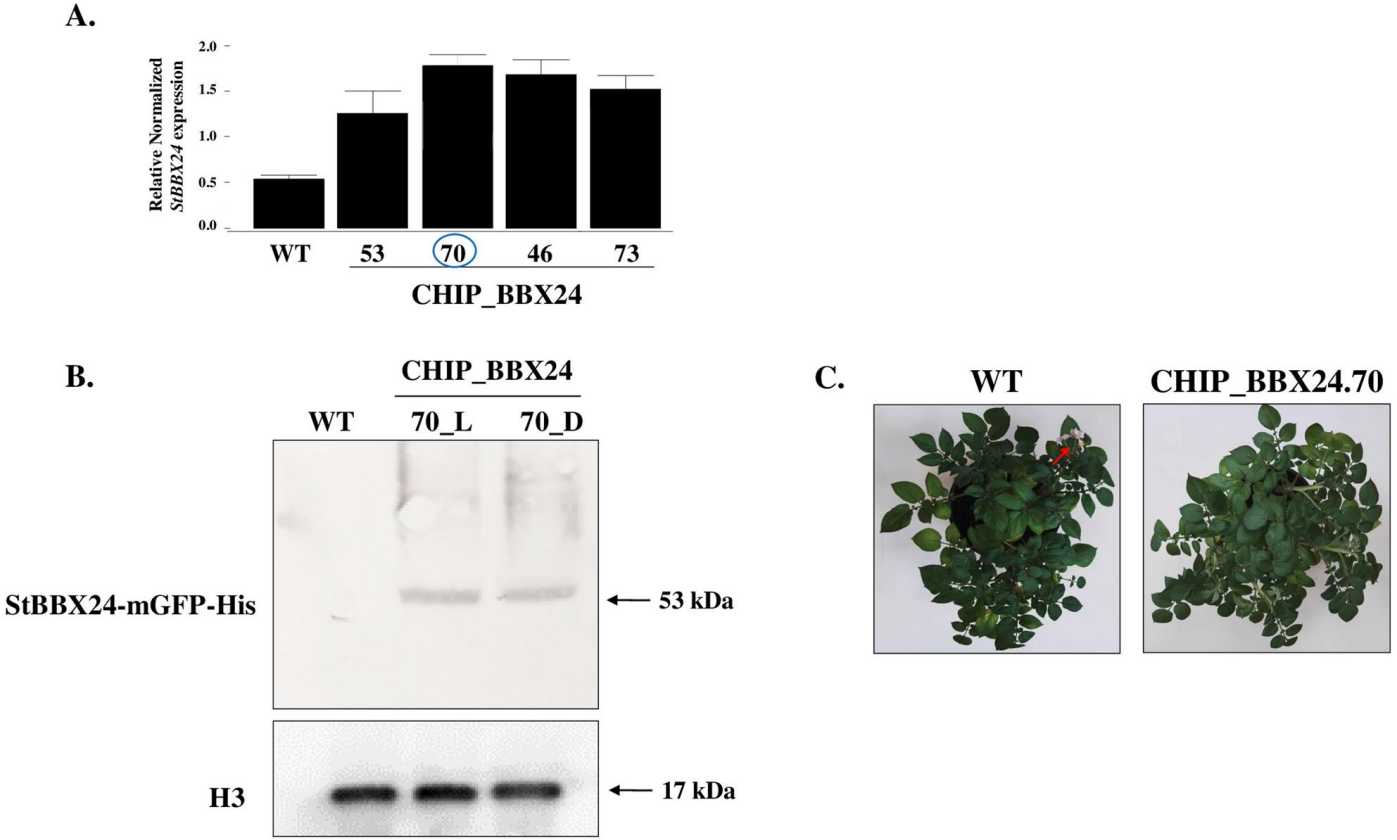
Phenotypic and molecular characterization of *S. tuberosum* CHIP-BBX24 transgenic lines. **A **Quantitative RT-PCR measurements of the* StBBX24* transcript abundance in selected transgenic lines (determined as a percentage of *StBBX24 *transcript level in the control potato plants). WT – wild type. **B** Western blot analysis of the presence of StBBX24-mGFP-His protein in the control Desirée plants (WT) and selected transgenic line: CHIP_BBX24.70 subjected to light (CHIP_BBX24.70_L) and dark (CHIP_BBX24.70_D) conditions. Western analysis of histone H3 abundance was performed using anti-H3 antibody (Agrisera) to ensure the purity of nuclear fraction and as an internal control. **C **Phenotype of *S. tuberosum* WT and CHIP_BBX24.70 transgenic line overexpressing *StBBX24* grown in standard conditions (20°C±1, 40 % relative humidity and PFD of 350 µmoles photons m^-2^ s^-1^) under a 14-h photoperiod for 57 days. The red arrow indicate the presence of open flowers

The StBBX24-mGFP-His transgenic lines were generated using Gateway recombination cloning technology and plant transformation vector pEarleyGate 103 containing double fusion C-terminal tags: mGFP and 6xHis. To create a Gateway entry clone, cDNA for *StBBX24* was amplified using Phusion™ High-Fidelity DNA polymerase (Thermo Scientific) and gene-specific primers (Table S1), cloned into pENTR™/SD/D-TOPO^®^ vector and sequenced. Individual transgenic lines were tested for *StBBX24* transcript level using RT-qPCR (Fig. 1A). Lines showing significantly increased *StBBX24* transcript abundance were analysed for the presence of the StBBX24-mGFP-His protein using Western blot method (Fig. 1B).

### *Solanum tuberosum* transformation

Vectors were introduced into *Agrobacterium tumefaciens* strain LBA4404. Plant transformation and regeneration were performed as described previously [41]. The transgenicity of the obtained plants was checked by PCR using specific primers. *StBBX24* expression level was assayed using quantitative real-time PCR and Western blot methods (Fig. 1 and Figures S1 A, B) [41]. Genomic DNA and total RNA were isolated from leaves using a GeneJET Plant Genomic DNA Purification Kit (Thermo Scientific) and “TRI Reagent” (Molecular Research Center, Inc.), respectively.

### Chromatin Immunoprecipitation (CHIP) assay and sequencing

10–15 g leaves of three-week-old *StBBX24*-mGFP-His transgenic line (CHIP_BBX24.70) collected at ZT6 (6 h of light) and ZT23 (9 h of dark) were cross-linked in 1% formaldehyde under a vacuum for 25 min. The fixation was stopped for 5 min by adding 0.125 M glycine. ~2.5 g of cross-linked leaves were ground to a powder in liquid nitrogen and subjected to nuclei isolation according to a modified method of Kaufmann et al. [46]. The homogenized tissue powder was suspended in a 25 mL buffer containing 10 mM sodium phosphate buffer, pH 7.0, 100 mM NaCl, 1 M 2-methyl 2,4-pentanediol and 10 mM β-mercaptoethanol supplemented with 1 cOmplete™ Protease Inhibitor Cocktail (Roche) tablet. Homogenate was filtered through the 53-µm polyester mesh (neoLab) and pelleted by centrifugation at 1000 *g* at 4 °C for 20 min. The nuclear pellet was resuspended five times in 5 mL buffer containing 10 mM sodium phosphate buffer, pH 7.0, 100 mM NaCl, 1 M 2-methyl 2,4-pentanediol, 10 mM β-mercaptoethanol, 10 mM MgCl_2_, 0.5% Triton X-100 supplemented with 1 cOmplete™ Protease Inhibitor Cocktail (Roche) tablet and centrifuged at 1000 *g* at 4 °C for 10 min. Finally, the pellet containing nuclei was resuspended in 0.5 mL ice-cold sonic buffer (10 mM sodium phosphate buffer, pH 7.0, 100 mM NaCl, 0.5% sarkosyl, 10 mM EDTA, pH 8.0, 1 cOmplete Protease Inhibitor Cocktail tablet) and transferred to Bioruptor 0.5 mL microtubes for chromatin shearing. The chromatin was sonicated using Bioruptor Plus with a refrigerated sonication bath (Diagenode, SA, Belgium) for 30 cycles of 30 s ON and 30 s OFF at a HIGH setting. 50 µL of sonicated chromatin was kept as an input control, while the remaining sonicated chromatin was used for immunoprecipitation. 3 µL of polyclonal anti-GFP(FL) antibody (Santa Cruz Biotechnology, Inc.) was used to immunoprecipitate the protein-DNA complex overnight at 4 °C, which was then purified using protein A agarose beads (Santa Cruz Biotechnology, Inc.). The precipitated DNA was recovered and verified by TapeStation HS D5000 Screen Tape and used for DNA library preparation with the TruSeq ChIP Library Preparation Kit (Illumina). The NovaSeq X 10B Sequencing System (Illumina) was applied for DNA sequencing with a 150 bp PE/SE configuration conducted by Macrogen Inc. (Seoul, Republic of Korea).

### CHIP-seq data processing and analysis

Raw reads were quality-analyzed using Trimmomatic software (version 0.40) [47]. All reads with a quality score of less than 20 (phred + 33) in a four-nucleotide window were trimmed from the ends of the reads. Reads shorter than 50 nucleotides were discarded from further analysis. Trimmed reads were mapped to the *S. tuberosum* reference genome [48] using the BWA algorithm (version 07.17) [49]. Optically duplicated reads were identified and removed from further analysis using Picard (version 2.20.5) (https://github.com/broadinstitute/picard). All biological replicates (BAM files) were merged into one file for each group. This step was performed using the systemPipeR software (version 1.6.2) and the mergeBamByFactor function [50]. Identification of peaks with the control/reference sample (input) was performed using the MACS2 callpeak algorithm (version 2.2.7.1) taking into account the peaks identified with statistical significance p(FDR) < 0.01 [51]. From the obtained peak coordinates, BED files were prepared using the bedtools getfasta program (version v2.31.1) [52] and the *S. tuberosum* reference genome. The identified peaks were annotated according to the information in the GFF file including the coordinates and names of the *S. tuberosum* genetic elements. The number of reads covering a given peak was counted for the identified and annotated peaks, and then a differential analysis was performed.

Statistical analysis was performed using the R software and the EdgeR algorithm (version 4.0) [53]. Functional analysis of statistically significant differential peaks (differential analysis, pFDR < 0.05) for two different variants: (i) dark-sample vs. dark-input sample and (ii) light-sample vs. light-input sample was performed using Interproscan (version 5.72) [54]. First, fasta sequences corresponding to statistically significant peaks were selected (differential analysis, pFDR < 0.05). Then, for selected statistically significant peaks, multiple functional annotations were assigned, taking into account data from MobiDBLite [55], ProSitePatterns [56], PANTHER [57], PRINTS [58], ProSiteProfiles [59], Interpro annotations, GO and Patways (MetaCyc [60]), Reactome [61]). Identification of DNA sequence motifs was performed using MEME-Chip (version 5.5.7) [62]. The most probable three conserved motifs were generated for each experimental variant. The detected motifs were subjected to comparative analysis using the TomTom program [63] (https://meme-suite.org/meme/tools/tomtom) and the JASPAR [64] CORE Plants and Arabidopsis database, and GO annotation using the GOMo program [63] (https://meme-suite.org/meme/tools/gomo) and the *Arabidopsis thaliana* (Plant) database.

### RNA-seq library preparation, sequencing and data analysis

Transcriptome analysis of the apical shoot parts and stolons in *StBBX24*-silenced and -overexpressed potato lines and WT plants was carried out using the RNA-seq method. The experiment was performed using two biological replicates. Each replicate consisted of 5- and 7-week-old apical shoot parts (Figure S2) and 4-week-old stolons (Figure S3) samples collected at ZT6. Total cellular RNA was isolated using TRI Reagent^®^ RT (Molecular Research Center, Inc.) and treated with DNase I during RNA purification. The quality and quantity of RNA were verified using a DS-11 FX spectrophotometer (DeNovix Inc.). RNA integrity number (RIN) of samples and rRNA ratio were checked using Bioanalyzer RNA Nano 6000 chip (Agilent Technologies, Inc.). Samples with RINs with scores ≥ 7 and rRNA ratio ≥ 1.7 were taken for sequencing. cDNA libraries were prepared using TruSeq Stranded mRNA LT Sample Prep Kit (Illumina) and sequenced using an Illumina platform NovaSeqX with a 2 × 150 bp PE configuration by Macrogen Inc. (Seoul, Republic of Korea).

To leverage all available data regarding the *S. tuberosum* reference genome, available annotations from previous projects (Potato Genome Sequencing Consortium; [65]) were stepwise merged with the most recent version DM8 [66] using LiftOff (1.6.3 version; [67]) at threshold. DM3 (Potato Genome Sequencing Consortium et al. [68]) and DM6 [65] contigs were mapped to DM8 reference using minimap2 (2.28-r1209; [69]) alignments between assemblies calculated at -x asm10 preset. Older annotations were transferred over assuming percentage identity (over 90%, -s 0.9 parameter in LiftOff), matching protein reference and presence of correct start and stop codons. In case of overlaps, the most recent annotation was kept and alternate identifiers were kept for reference (i.e. annotation of differential expression results). Final structural annotation (51883 genes, 54041 transcript isoforms) in GFF3 format is included in Additional Materials to this paper. Functional annotation was obtained using EnTAP (1.1.1; [70]) searches with DIAMOND (2.1.8; [71]) versus NCBI/RefSeq Plants, UniProt/SwissProt (downloaded at 23/05/2024), as well as eggnog-mapper (2.1.12; [72]) using EggNOG 5.0 orthologous group database [73].

The nf-core [74] RNA-seq pipeline version 3.15 was used to process Illumina sequencing results. Briefly, the short reads were trimmed with TrimGalore 0.6.7 and cutadapt 3.4 at pipeline default settings. Subsequently, these were processed using three separate approaches to mapping and quantification: RSEM (1.3.1; [75]) + STAR (2.7.10a; [76]), StringTie (2.2.1; [77]) + STAR and kallisto (0.48; [78]). Results of all three methods were further imported into R with tximport (1.30.0), normalised taking into account transcript lengths and modelled using DESeq2 (1.42). Expression was treated as a two-factor (genotype and time) generalised linear model including interaction terms. Applicable likelihood ratios were calculated per gene, based on reduced models omitting tested terms. In pairwise comparisons, genes with the base mean expression of at least 5, |log2(Fold Change)| of at least 2, and corrected P- value less or equal to 0.01 were reported as significantly differentially expressed. Gene Ontology term representation analysis was conducted with topGO (2.54; [79]) using the Fisher exact test (at 0.05 significance threshold). Parent-child weighting algorithm [80] was selected to take into account inheritance relationships in the GO term hierarchy.

### Real-time quantitative PCR (RT-qPCR)

Total RNA was isolated from the leaves of WT plants and transgenic lines using “TRI Reagent” (Molecular Research Center, Inc.) according to the product manual. The RNA was treated with RNase-free DNaseI (Ambion) and purified by phenol*-*chloroform extraction and ethanol precipitation. Sample concentration was measured using a DS-11 FX spectrophotometer (DeNovix Inc.). The Maxima Reverse Transcriptase (Thermo Scientific) was used for cDNA synthesis from 2 µg of purified RNA template. Real-time qPCR was performed using a CFX96 Touch^TM^Real-Time PCR detection system (Bio-Rad, Hercules, USA) and Maxima SYBR Green/ROX qPCR Master Mix (Thermo Scientific). Each assay using gene-specific primers (Table S1) amplified a single product of the appropriate size with high PCR efficiency. The fluorescence data analysis was conducted using the CFX^™^ Software (Version 3.0, Bio-Rad, Hercules, USA). The normalized expression of the target gene (ΔΔCq) was calculated as the relative quantity of the target normalized to the quantity of the *StEF-1-α* reference gene [41]. The values presented are the means of two technical replicates from four independent biological samples.

### Protein extracts preparation

Nuclei-enriched fractions were isolated from 2-week-old *S. tuberosum* leaves as described by Kiełbowicz-Matuk et al. [81]. Samples were centrifuged at 13,000 *g* for 10 min to pellet the insoluble chromatin. The chromatin pellet was washed and resuspended in 50 mM Tris-HCl, pH 8.0, 30 mM NaCl, 1 mM PMSF, 1% sodium deoxycholate, 1% SDS, 0.5% Triton X-100, and incubated at 4 °C for 1 h before centrifugation at 14,000 *g* for 10 min. Proteins were precipitated with four volumes of acetone. Total protein concentration was measured using a modified Lowry method (Thermo Scientific).

### Western blot analysis

Proteins (10–40 µg) were separated in SDS-PAGE gels containing 13% acrylamide and then electroblotted onto a 0.22 μm nitrocellulose membrane (Schleicher and Schuell, Germany) using a semi-dry blotting apparatus (Bio-Rad). Membranes were stained with Ponceau red to ensure that equal protein amounts were transferred in each lane. The Western blot analysis was performed using the StBBX24 antiserum and anti-GFP(FL) antibody (Santa Cruz Biotechnology, Inc.) diluted 1:500. Antibody raised against histone H3 was purchased from Agrisera (Vännäs, Sweden) and used diluted 1:5000. Bound antibodies were detected using an anti-rabbit immunoglobulin G (horseradish peroxidase conjugate) 1:10,000 (Agrisera) and a chemiluminescent substrate (Western Bright Quantum, Advansta). The Western blotting was performed using a G: BOX Imaging System (Syngene). Analyses were carried out using protein extracts from two replicates and two independent sets of plants.

## Results

### Identification of StBBX24 DNA binding sites from CHIP-seq analysis

For a better understanding of the regulatory role of StBBX24 in flowering and tuberization in cultivated potato, the identification of target genes was performed using the CHIP-seq method. For this purpose, we studied the *StBBX24*-mGFP-His overexpressing potato lines (CHIP_BBX24.46, CHIP_BBX24.53, CHIP_BBX24.70, and CHIP_BBX24.73), and finally chose one line: CHIP_BBX24.70 for mapping the genome-wide binding sites of StBBX24 during the light (6 h of light, CHIP_BBX24_L) and dark (9 h of dark, CHIP_BBX24_D) phase (Fig. 1A-C).

The raw sequence data coming from high throughput sequencing was of good quality with a high surviving read percent (>90%) (Table S2) and was submitted to the NCBI SRA database. We mapped the sequencing reads of DNA fragments to the *Solanum tuberosum*.SolTub_3.0 reference genome using the BWA algorithm (version 07.17) [49]. We identified 105 395 337 and 102 254 115 uniquely mapped reads for light and dark conditions, respectively, which were distributed throughout the potato genome (Table S3).

Identifying peaks involving the input sample was performed using the MACS2 callpeak algorithm considering the peaks detected with statistical significance p(FDR) < 0.01. Overall, 134 369 binding peaks of StBBX24 were detected for dark and 135 162 for light, respectively, with a Q-value < 0.05 for the CHIP-seq data sets. The MACS2 algorithm identified peaks in a ‘*de novo’* mode with a ‘nomodel’ parameter, meaning that no reference model was used. The annotation with the *S. tuberosum* reference genome allowed the correct genomic coordinates to be assigned to chromosomal positions. The final number of peaks was 124 686 for dark and 125 764 for light conditions. Differential analysis was performed using R software and the EdgeR algorithm, distinguishing differential peaks in a statistically significant manner (pFDR < 0.05) for each of the indicated experimental conditions. Finally, 4598 statistically significant peaks were identified for light and 4971 for darkness (Additional Data Set 1 and 2). After calling the peak, we aimed to investigate the location of the peak across the whole genome.

**Fig. 2:**
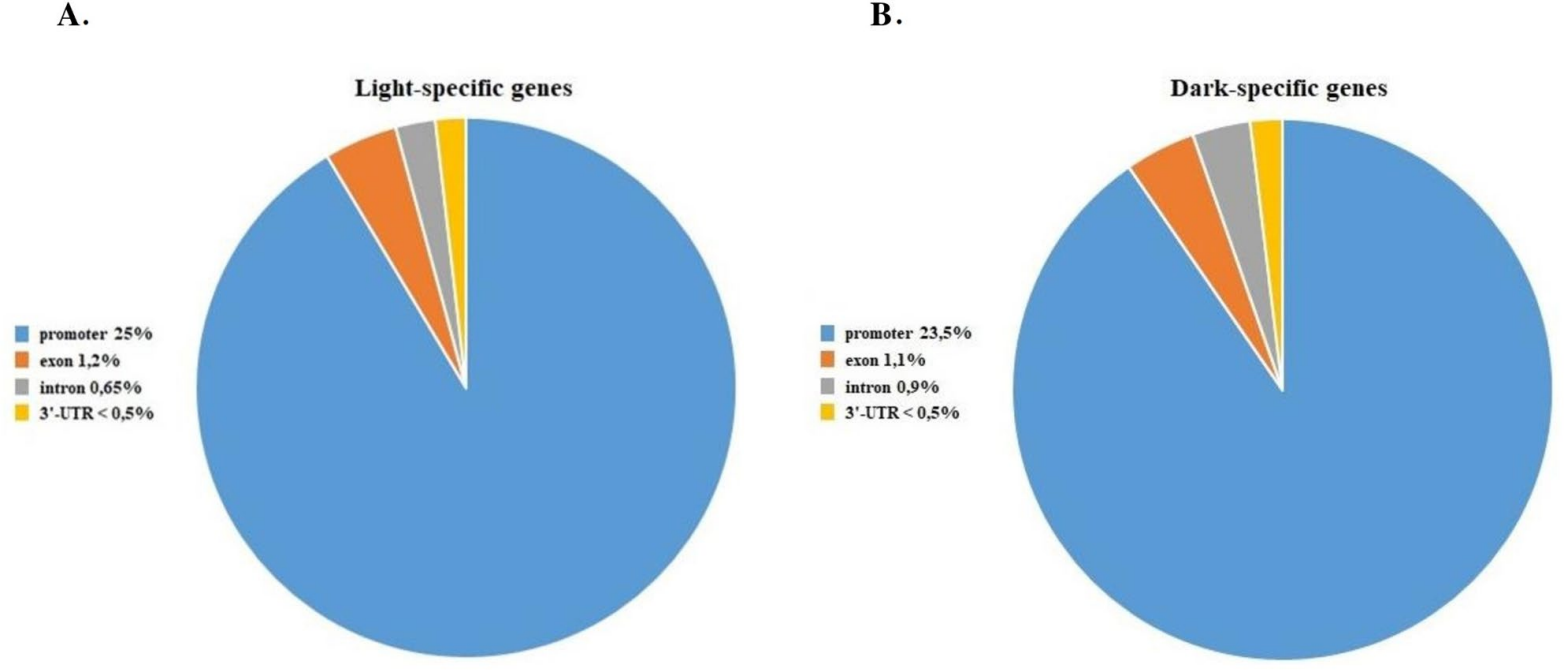
Distribution of peaks by genic regions of StBBX24 under light (**A**) and dark (**B**) conditions

In the first instance, we analyzed whether the peaks in the CHIP experiment were distributed by genic regions. We revealed that approximately 27% of the StBBX24-binding peaks were in the genic regions of 4598 light-specific genes. Among them, 25% were located in promoter regions (~ 3 kb to the TSS), 1.2% in exon regions, 0.65% in intron regions and less than 0.5% in 3′-UTRs (Additional Data Set 1, Fig. 2A). Similar results were obtained for darkness, about 26% of the peaks were identified in the intragenic regions of 4971 darkness-specific genes, including 23.5% located in promoter regions, 1.1% in exon regions, 0.9% in intron regions and less than 0.5% in 3′-UTRs (Additional Data Set 2, Fig. 2B). Under both experimental conditions, the intergenic regions represent more than 70% of the total binding sites and about 40% of the binding sites were not annotated (unknown).

### Binding motif sequence analysis reveals StBBX24 light and dark phase specific *cis*-regulatory elements

**Fig. 3:**
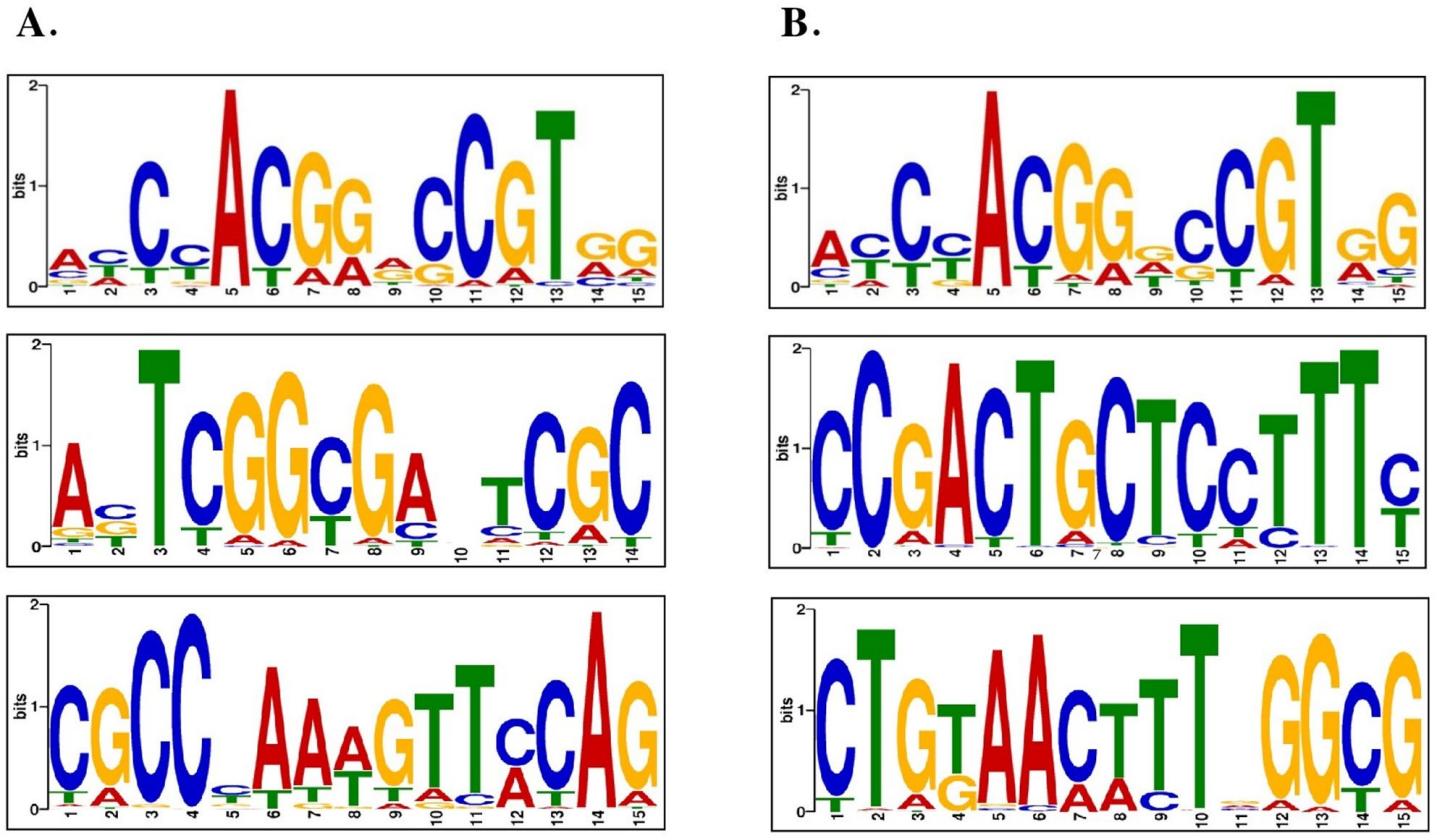
MEME-CHIP analysis of StBBX24 binding motifs. Three most representative motif patterns for light (**A**) and dark (**B**) conditions

To recognize the StBBX24 binding specific sequence patterns, 1-kb flanking sequences around the peak summits were analyzed by a motif-based sequence discovery tool MEME-CHIP (Fig. 3A and B). Using the MEME suite, the most likely three conservative motifs were found for light and dark. The most statistically relevant motifs for light are AHCYACGGRCCGTRG, ABTCGGCGAHTCGC and CGCCYAAWGTTMCAG, and for dark: AYCYACGGDCCGTRG, CCGACTGCTCCTTTY and CTGKAACTTTVGGCG. Interestingly, one motif ‘AY/HCYACGGD/RCCGTR’, is common to light and dark.

Then, identified motifs were compared against the JASPAR CORE Plants and Arabidopsis transcription factors binding profiles database using the TomTom tool (Tables 1 and 2). We found that the ‘AY/HCYACGGD/RCCGTR’ motif is a target for the transcription factors belonging to the Myb/SANT domain family, i.e., MYB15 (MA0574.1), bHLH (basic Helix-Loop-Helix) family i.e., UNE10 (MA1074.1) protein, TCP (TEOSINTE BRANCHED1/CYCLOIDEA/PROLIFERATING CELL FACTOR) family i.e., (MA1285.1) protein and AP2-EREBP (APETALA2-ETHYLENE-RESPONSIVE ELEMENT BINDING PROTEINS) family, i.e., RAP2-4 (MA1795.1). Another motif, ‘ABTCGGCGAHTCGC’ is recognized by the CBF1 transcription factor (AP2EREBP_tnt.CBF1_col_a_m1) belonging to the AP2-EREBP family. In contrast, the ‘CCGACTGCTCCTTTY’ dark-specific motif can be recognized by PHL7 (MA1384.1) and PHL12 (MA1166.1) proteins belonging to the G2-like transcription factor family and bZIP50 (bZIP_tnt.bZIP50_col_v31_m1) a member of the basic leucine zipper family. The ‘CGCCYAAWGTTMCAG’ motif not matched to any groups was considered unique.

**Table 1:** TomTom analysis for light conditions

Query_ID	Target_ID	*p*-value	E-value	q-value	Query_consensus	Target_consensus	Orientation
AHCYACGGRCCGTRG	MA0574.1	0.000458441	0.75	1	ATCTACGGGCCGTGG	CCACCTACCGTCG	-
AHCYACGGRCCGTRG	MA1021.1	0.00342991	5.63	1	ATCTACGGGCCGTGG	CCACGTGC	+
AHCYACGGRCCGTRG	MA1285.1	0.00401269	6.58	1	ATCTACGGGCCGTGG	GGTGGGACCCACGGCAAATGG	-
AHCYACGGRCCGTRG	MA1074.1	0.00435588	7.15	1	ATCTACGGGCCGTGG	CCACGTGC	+
AHCYACGGRCCGTRG	MA0941.1	0.00605587	9.94	1	ATCTACGGGCCGTGG	AACACGTGTCATG	-
ABTCGGCGAHTCGC	AP2EREBP_tnt.CBF1_col_a_m1	0.00523356	8.59	1	ATTCGGCGATTCGC	GATGTCGGCGAA	-

Although all matches returned q-values of 1 (*p*-values adjusted with the Bonferroni correction), results with low p-values and E-values are shown to retain biologically meaningful motif similarities

**Table 2:** TomTom analysis for darkness

Query_ID	Target_ID	*p*-value	E-value	q-value	Query_consensus	Target_consensus	Orientation
AYCYACGGDCCGTRG	MA0574.1	0.00080088	1.31	1	ACCCACGGGCCGTGG	CGACGGTAGGTGG	+
AYCYACGGDCCGTRG	MA1285.1	0.00437158	7.17	1	ACCCACGGGCCGTGG	GGTGGGACCCACGGCAAATGG	-
AYCYACGGDCCGTRG	TCP_tnt.At2g45680_col_b_m1	0.00437158	7.17	1	ACCCACGGGCCGTGG	GGTGGGACCCACGGCAAATGG	-
AYCYACGGDCCGTRG	TOE2_2	0.00494447	8.11	1	ACCCACGGGCCGTGG	AACCTACG	+
AYCYACGGDCCGTRG	MA1795.1	0.00547521	8.98	1	ACCCACGGGCCGTGG	ATGGTCGGTG	-
AYCYACGGDCCGTRG	MA1021.1	0.00580287	9.52	1	ACCCACGGGCCGTGG	CCACGTGC	+
CCGACTGCTCCTTTY	MA1166.1	0.00387882	6.37	1	CCGACTGCTCCTTTT	GGAATATTCCCTTT	-
CCGACTGCTCCTTTY	G2like_tnt.At3g12730_col_a_m1	0.00387882	6.37	1	CCGACTGCTCCTTTT	GGAATATTCCCTTT	-
CCGACTGCTCCTTTY	MA1384.1	0.00404132	6.63	1	CCGACTGCTCCTTTT	GGAATATTCTTTT	-
CCGACTGCTCCTTTY	G2like_tnt.At2g01060_col_m1	0.00404132	6.63	1	CCGACTGCTCCTTTT	GGAATATTCTTTT	-
CCGACTGCTCCTTTY	bZIP_tnt.bZIP50_col_v31_m1	0.00461528	7.57	1	CCGACTGCTCCTTTT	TGATGACGTCATCTTTT	+
CCGACTGCTCCTTTY	C2H2_tnt.TF3A_col_a_m1	0.00609274	10	1	CCGACTGCTCCTTTT	CCTCCTCCTCCTCCTCCTC	+

Although all matches returned q-values of 1 (*p*-values adjusted with the Bonferroni correction), results with low p-values and E-values are shown to retain biologically meaningful motif similarities

### StBBX24 directly targets other transcriptional regulatory elements

To identify targets associated with the transcription regulation mediated by StBBX24 during the day-night cycle, the genes associated with each peak encoding transcription factors (TF), chromatin regulators (CRs) and transcriptional regulators (TRs) were verified with the Online Plant Transcription Factor and Transcriptional Regulator Categorization and Analysis Tool [82].

We revealed that 138 regulatory genes are regulated by StBBX24 in the light including TFs (85%), CRs (11.5%) and TRs (3.5%). In comparison, 126 regulatory genes are targeted by StBBX24 in the darkness, including TFs (86.4%), CRs and TRs (6.8% each) (Table S4). Among these, 60 genes were common for both light and darkness. We found that the identified putative transcription factors are members of multiple families, with the largest number of genes belonging to the C2H2, MADS-type I, MYB-HB and AP2-EREBP. In further studies, we focused on genes encoding the TFs with peaks within promoter regions (~ 3 kb to TSS) (Table 3). We classified them into three groups, i.e., light-specific genes, dark-specific genes and genes occurring in both light and darkness.

**Table 3:** Transcription factors targeted by StBBX24 under light and dark conditions

Light-specific		Dark-specific		Light and dark specific	
Gene ID	TF family	Gene ID	TF family	Gene ID	TF family
PGSC0003DMG400007174	MYB-HB-like	PGSC0003DMG400009276	TCP	PGSC0003DMG400026332	NAM
PGSC0003DMG400034516	MYB-HB-like	PGSC0003DMG400043978	MADS-type1	PGSC0003DMG400025355	ssDNA-binding
PGSC0003DMG400017813	AP2-EREBP	PGSC0003DMG400000278	C3H	PGSC0003DMG400028638	MADS-type1
PGSC0003DMG400000441	C2C2-Dof	PGSC0003DMG400030389	MYB-HB-like	PGSC0003DMG400012599	C3H
PGSC0003DMG400015565	Hap3/NF-YB	PGSC0003DMG400025976	bHLH	PGSC0003DMG400039175	WRKY
PGSC0003DMG400017761	C2H2	PGSC0003DMG400000062	MADS-type1	PGSC0003DMG402008471	WD40-like
PGSC0003DMG400010912	C2H2	PGSC0003DMG400001386	C2H2	PGSC0003DMG400002098	C2H2
PGSC0003DMG400022689	MYB-HB-like	PGSC0003DMG400023582	HD-ZIP	PGSC0003DMG400006209	MYB-HB
PGSC0003DMG401006284	SBP	PGSC0003DMG400018439	MYB-HB	PGSC0003DMG400036493	AP2-EREBP
PGSC0003DMG400029773	Lambda-DB	PGSC0003DMG400035463	MADS-type1		
PGSC0003DMG400015498	C2H2	PGSC0003DMG400023295	AP2-EREBP		
PGSC0003DMG400012653	AS2-LOB	PGSC0003DMG400046191	WD40-like		
PGSC0003DMG400039636	Hap3/NF-YB				
PGSC0003DMG402015259	Hap3/NF-YB				
PGSC0003DMG400039820	bZIP				
PGSC0003DMG400026068	C2H2				
PGSC0003DMG400004470	MYB				
PGSC0003DMG400036318	B3-domain				

### StBBX24 binds to genes involved in floral induction and flower development

We earlier reported that the the *StBBX24* gene expression was differentially regulated depending on organ type during vegetative growth and reproductive development [42]. Moreover, we demonstrated that the StBBX24 protein deficiency resulted in flowering acceleration in potato [42].

**Fig. 4:**
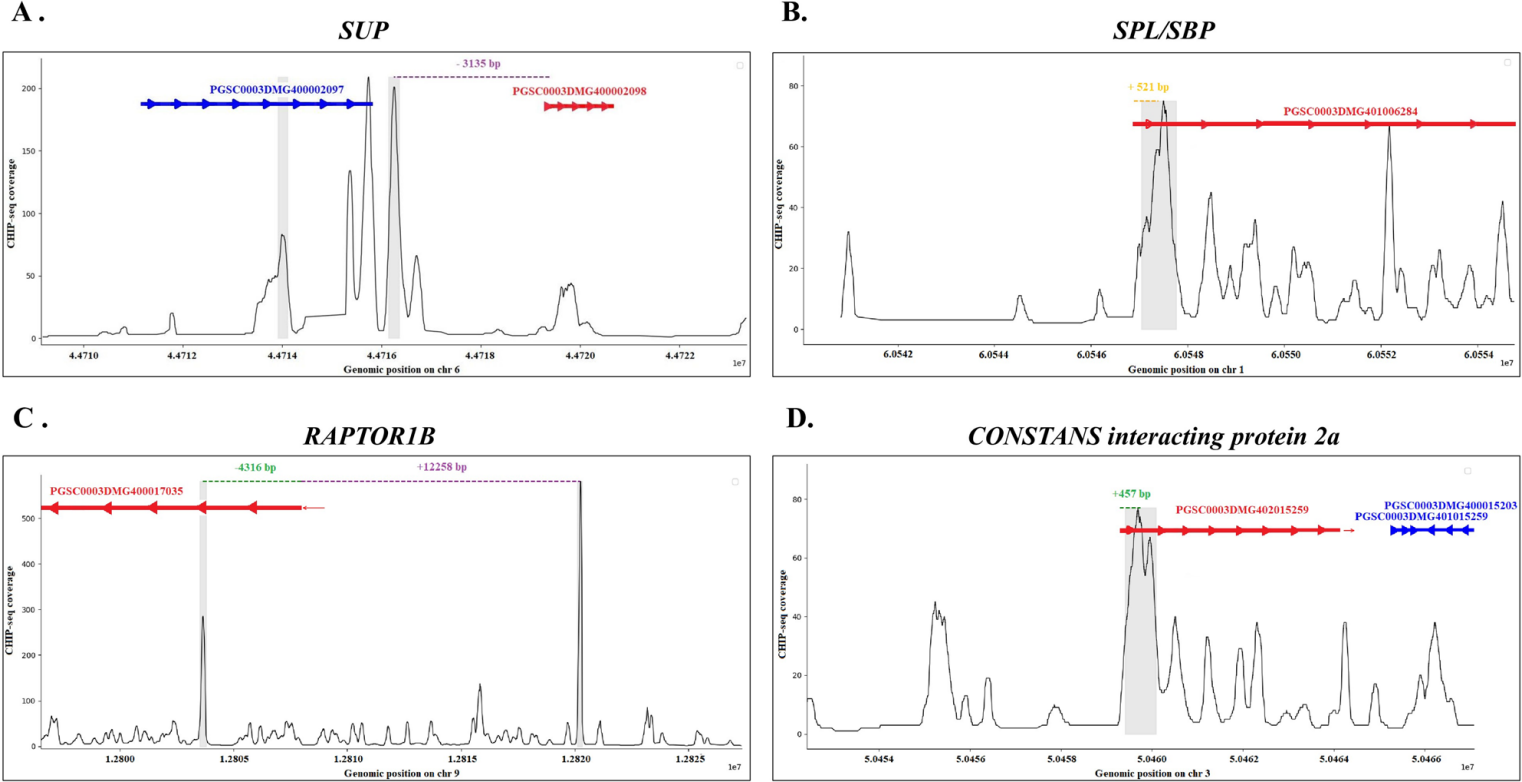
CHIP-seq reveals direct StBBX20 targets. Normalized ChIP-seq coverage (y-axis) plotted across genomic coordinates (x-axis) for four representative loci: *StSUP* (**A**), *StSPL/SBP* (**B**), *StRAPTOR1B* (**C**)and *StCONSTANS interacting protein 2a *(**D**). Each graph covers a 7 kb genomic window centered on the peak region. Genes are shown as horizontal arrows: red = annotated gene, blue = other neighbouring genes. Arrows indicate transcriptional orientation, and represent exons (connected by thin lines representing introns). Shaded grey boxes highlight significant ChIP-seq peaks. Dashed vertical lines (green or yellow) indicate the distance (in bp) between the peak summit and the annotated transcription start site (TSS) of the nearest gene

To further dissect the molecular mechanisms underlying the role of StBBX24 in potato reproductive growth, we focused on the identification of the genes determining the flowering time and floral development (Fig. 4A-D). We found that two light-specific StBBX24-binding regions were positioned < 1 kb upstream of the transcription start sites (TSS) for *CONSTANS interacting protein 2a* (ID: PGSC0003DMG402015259) and *StSPL/SBP* (*SQUAMOSA Promoter-Binding Protein-Like*) (ID: PGSC0003DMG401006284), being orthologues of the *NF-YC1* (*HAP5*) and *SPL7* genes in Arabidopsis (75.65% and 44.62% of identity, respectively). Another putative light-mediated target is located 3.7 kb upstream of the TSS of *StHY5* gene (*ELONGATED HYPOCOTYL 5*) (ID: PGSC0003DMG400039820). Interestingly, several direct targets of StBBX24 were identified both for the light and the dark conditions, including *StRAPTOR1B* (*REGULATORY ASSOCIATED PROTEIN of TARGET OF RAPAMYCIN*) (ID: PGSC0003DMG400017035) gene encoding protein involved in promoting flower transition [83], *StMADS47* (ID: PGSC0003DMG400000062) and *StMADS153* (ID: PGSC0003DMG400028638) genes [84] positioned upstream of TSS and *StSUP* gene (*SUPPERMAN*) (ID: PGSC0003DMG400002098) encoding a repressor of floral meristem determinacy localized ~ 3 kb downstream of TSS. We also paid attention to the StBBX24 binding peaks located between 3 and 8.6 kb downstream of TSS representing the flowering pathway genes, i.e., *StSPL3* (*SQUAMOSA PROMOTER BINDING PROTEIN-HOMOLOGUE 3*) (ID: PGSC0003DMG400029162), *StSOC1* (SUPPRESSOR OF OVEREXPRESSION OF CONSTANS1), (ID: PGSC0003DMG401024252), *StMADS61* (ID: PGSC0003DMG400038730) and *StFPF1* (*FLOWERING PROMOTING FACTOR-LIKE 1*) (ID: PGSC0003DMG400011345) for which the role of StBBX24 protein as a distal transcriptional regulator cannot be excluded.

**Fig. 5:**
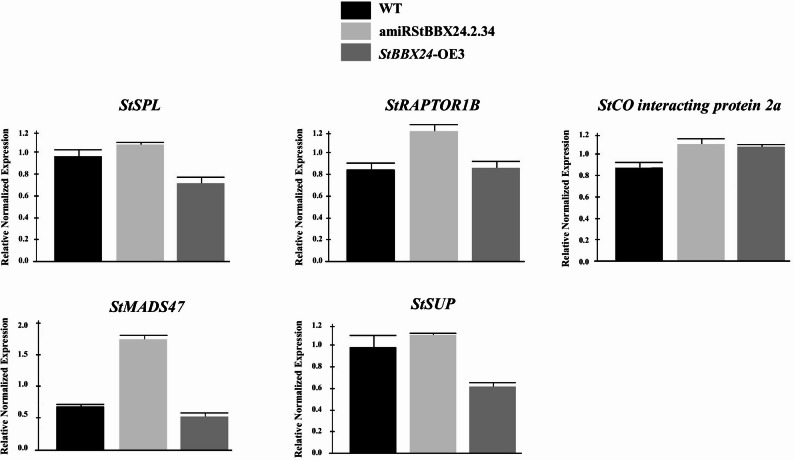
Effects of *StBBX24* silencing and overexpression on select target genes in *S. tuberosum* transgenic lines. Quantitative RT-PCR analysis of *CONSTANS interacting protein 2a*, *StSPL/SBP*, *StRAPTOR1B*, *StMADS47 *and *StSUP* transcript levels in leaves of three-week-old WT plants, and amiRStBBX24.2.34 and *StBBX24*-OE3 transgenic lines under a 14-h photoperiod during the light and dark phases. RT-qPCR analyses were normalized using the threshold cycle (C1) values corresponding to the *StEF-1-α* reference genes. The normalized expression of the target gene (ΔΔCq) was calculated as the relative quantity of target normalized to the quantities of the reference gene according to the manufacturer’s software. The values presented are the means of two technical replicates from four independent biological samples

To validate the effects of *StBBX24* sillencing and overexpression on select target genes activity, we measured the transcript abundance of five selected targets, i.e., *CONSTANS interacting protein 2a*, *StSPL/SBP*,* StRAPTOR1B*, *StMADS47* and *StSUP* in leaves of three-week-old *S. tuberosum StBBX24*-silenced and -overexpressed transgenic lines (amiRStBBX24.2.34 and *StBBX24-*OE3) relative to the WT plants by RT-qPCR (Fig. 5). We observed elevated transcript levels of *StMADS47* and *StRAPTOR1B* in the amiRStBBX24.2.34 line compared to the WT and *StBBX24-*OE3 line, while the levels of *StSPL/SBP* and *CONSTANS interacting protein 2a* were not significantly different from the WT (Fig. 5). Interestingly, we also found a substantial down-regulation of *StSUP* gene expression in *StBBX24-*OE3 line as compared with in WT.

Altogether, identifying the CHIP-seq candidate genes provided a more comprehensive understanding of the molecular basis of potato flowering and the participation of the StBBX24 protein in this process.

### StBBX24 regulates the transcription of genes involved in the flowering pathway

**Fig. 6:**
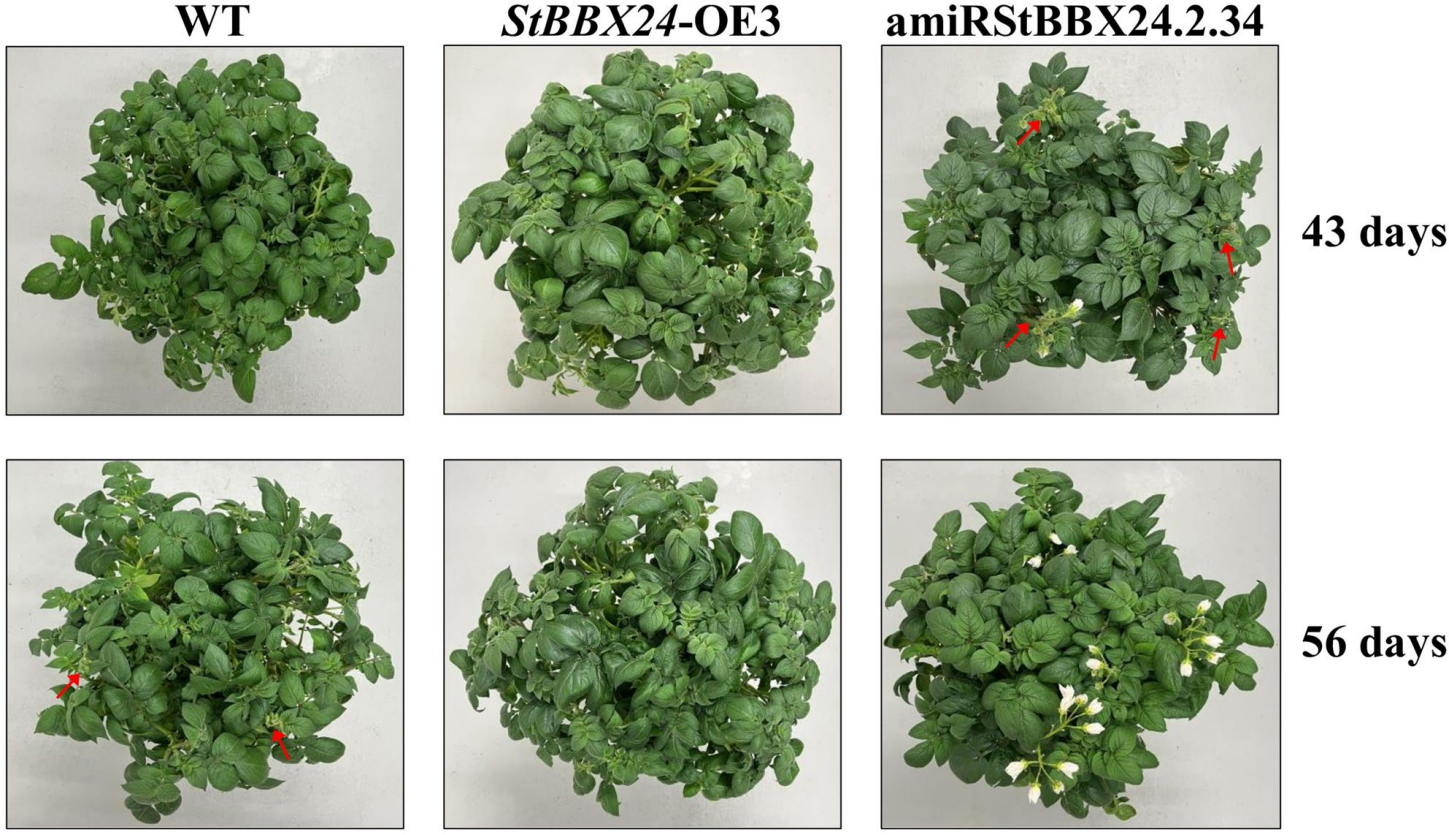
Flowering phenotype of *S. tuberosum* plants *StBBX24*-silenced and overexpressed. Phenotype of WT, amiStRBBX24.2.34 and *StBBX24*-OE3 transgenic plants at the pre-flowering and flowering stages. Twenty plugs from potato tubers per genotype were grown in plastic pots in the growth room in standard conditions under a 14-h photoperiod. The red arrows indicate the presence of flower buds and open flowers. WT – wild-type

We previously revealed the early flowering phenotype of *StBBX24*-silenced potato plants [41], which is consistent with our present observation. We noticed that the lack of StBBX24 protein strongly promoted flowering in the amiRStBBX24.2.34 line, as compared with WT andplants overexpressing *StBBX24* (Fig. 6).

To identify other genes related to the flowering time and floral development, we expanded our research on apical shoot parts transcriptome in *S. tuberosum* wild-type plants and *StBBX24*-silenced and -overexpressed transgenic lines (amiRStBBX24.2.34 and *StBBX24-*OE3). The apical shoot part, in particular the shoot apical meristem, fulfils a major role in flowering via the initiating of this process and directing the development of floral structures. Plants at two different developmental stages: 5- and 7-week-old were taken for analysis (Figure S2).

**Fig. 7:**
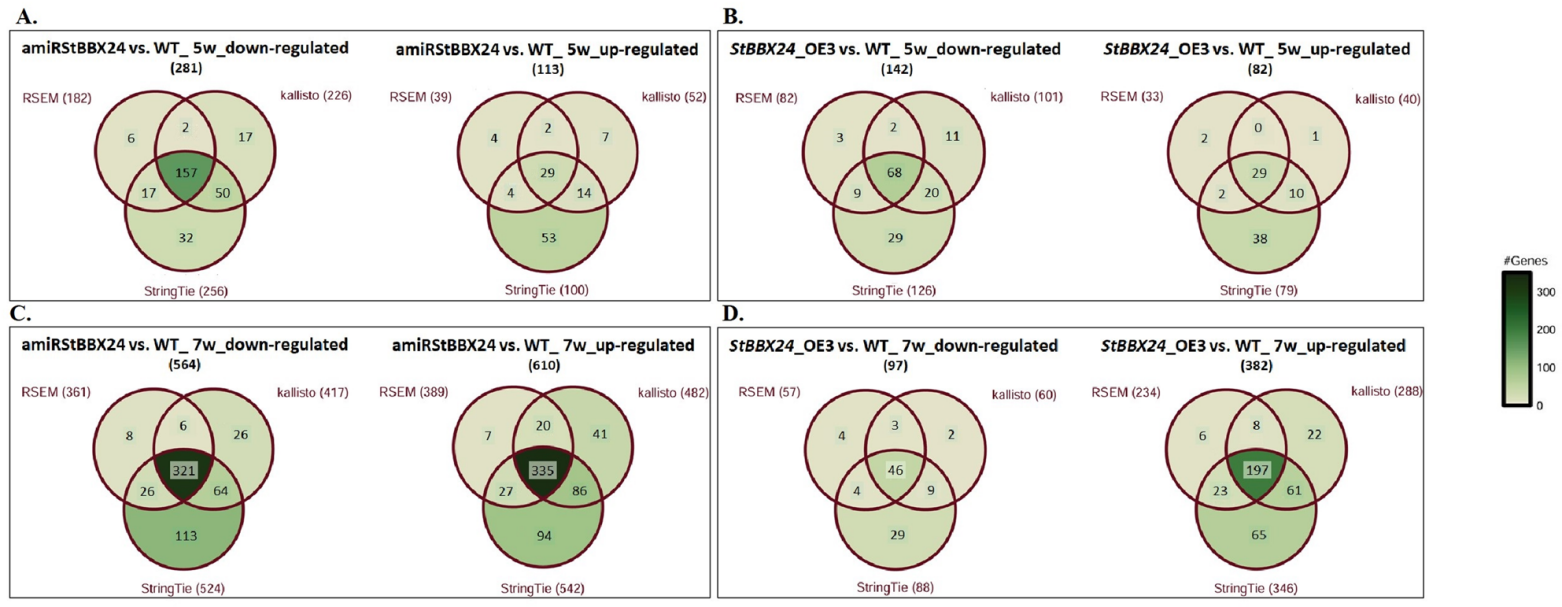
Venn diagrams representing the number of differentially expressed genes in apical shoot parts of 5-week-old amiRStBBX24.2.34 (**A**) and StBBX24-OE3 (**B**) transgenic lines and 7-week-old amiRStBBX24.2.34 (**C**) and *StBBX24*-OE3 (**D**) transgenic lines using three separate approaches RSEM, StringTie and kallisto

To exploit all available data on the *S. tuberosum* reference genome, annotations from previous projects were progressively merged with the latest version (Additional Data Set 3). Quality statistics of raw sequencing data and analysis of the alignment of processed data to the reference genome sequence were included in Table S5. Final structural annotation was included in Additional Data Set 4. Three separate approaches, i.e., RSEM + STAR, StringTie + STAR and kallisto were applied for mapping and quantification (Additional Data Set 5 and 6). To comprehend how gene expression alters in time and how various genetic backgrounds influence these changes, the gene expression levels were modelled using a generalized linear model (GLM) considering two key factors: genotype and time (Fig. 7A-D).

When contrasting transcript levels at two development stages of apical shoot parts, we revealed that the total number of DEGs identified in the amiRStBBX24.2.34 transgenic line was significantly higher in 7-week-old than in 5-week-old *S. tuberosum* plants (Fig. 7A and C). Comparisons of up-regulated and down-regulated genes using three separate approaches (RSEM + STAR, StringTie + STAR and kallisto) revealed that the common number of down-regulated genes was 157 and 321 in 5- and 7-week-old plants, respectively, with the number of up-regulated genes being approximately eleven times higher at a later stage than at an earlier stage of apical shoot development. Similarly, in transgenic lines overexpressing *StBBX24*, the total number of down- and up-regulated genes was about two times lower in 5-week-old plants compared to 7-week-old plants, whereby the most significant difference was observed in the number of up-regulated genes in two stages of *StBBX24*-OE3 line development (a 6-fold increase in expression of DEGs after 7 weeks compared to 5 weeks) (Fig. 7B and D). It is worth noting that the highest number of all DEGs was identified using the StringTie while the lowest using the RSEM method, regardless of the differentiating factor.

**Fig. 8:**
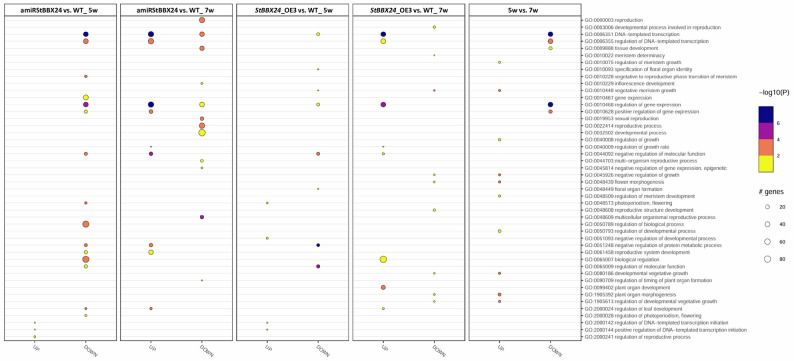
GO functional enrichment of differentially expressed genes in 5- and 7-week-old apical shoot parts of *StBBX24*-silenced and -overexpressed lines. The sizes of the wheels indicate the number of genes enriched in the GO term. The color of the wheels represents the Q-value

To provide a functional classification for the identified DEGs, the Gene Ontology (GO) database was applied. We found that in the category ‘biological process’, several terms of interest were enriched including regulation of biological process (GO:0050789), photoperiodism, flowering (GO:0048573), vegetative to reproductive phase transition of meristem (GO:0010228), regulation of photoperiodism, flowering (GO:2000028), reproductive process (GO:2000241), reproductive system development (GO:0061458), reproduction (GO:0000003), developmental process (GO:0032502), DNA-templated transcription (GO:0006351), positive regulation of gene expression (GO:0010628), regulation of gene expression (GO:0010468) and regulation of DNA-templated transcription (GO:0006355) (Fig. 8).

Following the classification of the mode of action of differently expressed genes and based on the delayed flowering of silenced lines and lack or deleted flowering of overexpressed lines, we paid more attention to genes associated with the flowering pathway at two different stages of shoot apices development.

In the 5-week-old *StBBX24*-silenced line, we noticed decreased expression levels of *StCOL3/StBBX3* (PGSC0003DMG400027475), while the expression of the floral inducer *StSP6A* (PGSC0003DMG400023365) was up-regulated by a factor of 10.5. Meanwhile, in the 7-week-old silenced line, we observed significantly decreased expression levels of the *StBEL34* (PGSC0003DMG400008057), *StLSH1 *(*LIGHT-DEPENDENT SHORT HYPOCOTYLS 1*) (PGSC0003DMG400023406), *StKNOX10 *(*KNOTTED1-LIKE HOMEOBOX*) (PGSC0003DMG400004953) and *StSPL9* (*SQUAMOSA PROMOTER BINDING PROTEIN-LIKE 9*) (PGSC0003DMG400011433). Additionally, we found that among the up-regulated genes were Floral homeotic protein *AGAMOUS* (PGSC0003DMG400028442) and MADS-box transcription factor *StFBP4FBP4 *(*FLORAL BINDING PROTEIN 4*) (PGSC0003DMG400001938).

On the contrary, in 5-week-old *StBBX24* overexpressing lines, we noticed a decrease in the transcript level of some genes encoding a MADS-box transcription factor involved in flower development, including Floral homeotic protein AGAMOUS (PGSC0003DMG400028442), StSEP1 (SEPALLATA 1) (PGSC0003DMG400001376) and StFBP1 (PGSC0003DMG402007392). Meanwhile, the transcript amount for *StMYB84* (PGSC0003DMG400014550), an ortholog of the *MYB62* gene in Arabidopsis, which is involved in the supersession of *SUPERMAN* and *SOC1* genes expression, was up-regulated in the 7-week-old *StBBX24-OE3* line.

**Fig. 9:**
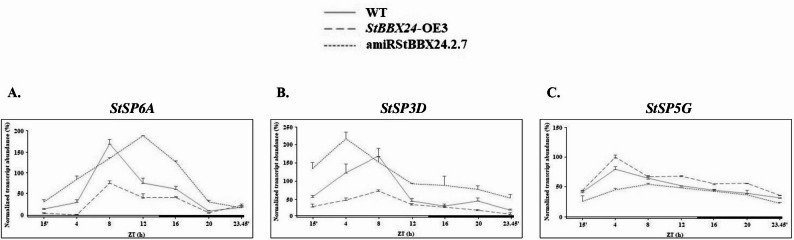
Expression profiles of flowering-related genes: *StSP6A *(**A**) (ID: PGSC0003DMG400023365/Soltu.DM.05G026370/DM8C05G26810), *StSP3D* (**B**)(ID: Soltu.DM.03G011110/DM8C03G11540) and *StSP5G* (**C**) (ID: Soltu.DM.05G0240301/DM8C05G24610) in 5-week-old *S. tuberosum* *StBBX24*-silenced (amiRStBBX24.2.34) and -overexpressed (*StBBX24*-OE3) lines, and WT plants using quantitative RT-PCR. The plant materials were collected at different time points (15′, 4, 8, 12, 16, 20, 23h45′) for 24 h under a 14-h photoperiod. ZT refers to the experimental time where the ZT0 point corresponds to light-on. The bars represent the subjective light and night conditions. RT-qPCR analyses were normalized using the threshold cycle (C1) values corresponding to the *StEF-1-α *reference gene. The normalized expression of the target gene (ΔΔCq) was calculated as the relative quantity of the target normalized to the quantities of the reference gene. The values presented are the means of two technical replicates from four independent biological samples. WT – wild type

In *S. tuberosum*, the contribution of FLOWERING LOCUS T-like (FT-like) proteins, including StSP6A and StSP3D as inducers and StSP5G as a repressor of floral initiation has been reported. Since *FT-like* genes are circadian-regulated via the CONSTANS/StBBX1 protein, to analyze changes in their transcript levels in apical shoots of *StBBX24*-silenced and -overexpressed plants, we additionally investigated the expression patterns of *StSP6A*, *StSP3D* and *StSP5G* genes using RT-qPCR (Fig. 9A-C). We revealed that in the amiRBBX24.2.34 silenced line, *StSP6A* and *StSP3D* transcript levels were substantially up-regulated as compared with WT (Fig. 9A and B), while *StSP5G* expression was significantly decreased (Fig. 9C). Substantial changes in transcript accumulation were also observed in plants with *StBBX24* overexpression (*StBBX24*-OE3 line) compared with non-transformed plants. We observed that the transcript of *StSP6A* and *StSP3D* genes were down-regulated (Fig. 9A and B), whereas *StSP5G* expression significantly increased (Fig. 9 C).

### Transcriptome profiling of *StBBX24****-***silenced and -overexpressed potato plants identified genes potentially associated with tuberization

Our earlier studies showed that *S*. *tuberosum StBBX24-*silenced lines are characterised by an increased number of tubers compared to lines with *StBBX24* overexpression and wild-type plants [41]. The phenotypic analysis of the new generated amiRStBBX24.2.37 line confirmed previous findings (Table 4).

**Table 4:** Tubers yield (shape index, weight and the number) in *S. tuberosum* WT plants and *StBBX24*-silenced and overexpressed lines

Phenotype	Tubers shape index	Tuber weight [g]	The number of tubers
WT	1.39 (± 0.22)	18.82 (± 3.29)	60
*StBBX24*-OE3	1.48 (± 0.17)	17.07 (± 4.15)	54
amiRStBBX24.2.37	1.32 (± 0.12)	19.11 (± 3.09)	76

± Standard deviation (SD). Data represent the average of five pots (twenty plants) for each line

According to these data, we executed a large-scale screening for searching differentially expressed genes involved in StBBX24-mediated tuberization at the initial stage of tuber formation – hook stage in *StBBX24*-silenced and -overexpressed plants (Additional Data Set 7 and 8).

**Fig. 10:**
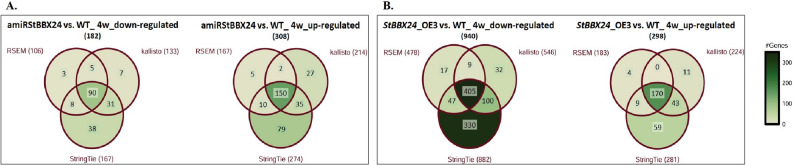
Venn diagrams representing the number of differentially expressed genes in 4-week-old stolons of WT, *StBBX24*-silenced (**A**) and -overexpressed (**B**) S. *tuberosum *plants using three separate approaches RSEM, StringTie and kallisto. WT – wild type

We identified 490 genes with a significant change in their expression level, more than 2.0-fold, in the *StBBX24-*silenced line. Among them, 308 were up-regulated and 182 were down-regulated (Fig. 10A). Meanwhile, in the *StBBX24*-OE3 line, the number of DEGs was 1236, with a significant predominance of down-regulated genes (940) compared to the number of up-regulated genes (296) (Fig. 10B).

**Fig. 11:**
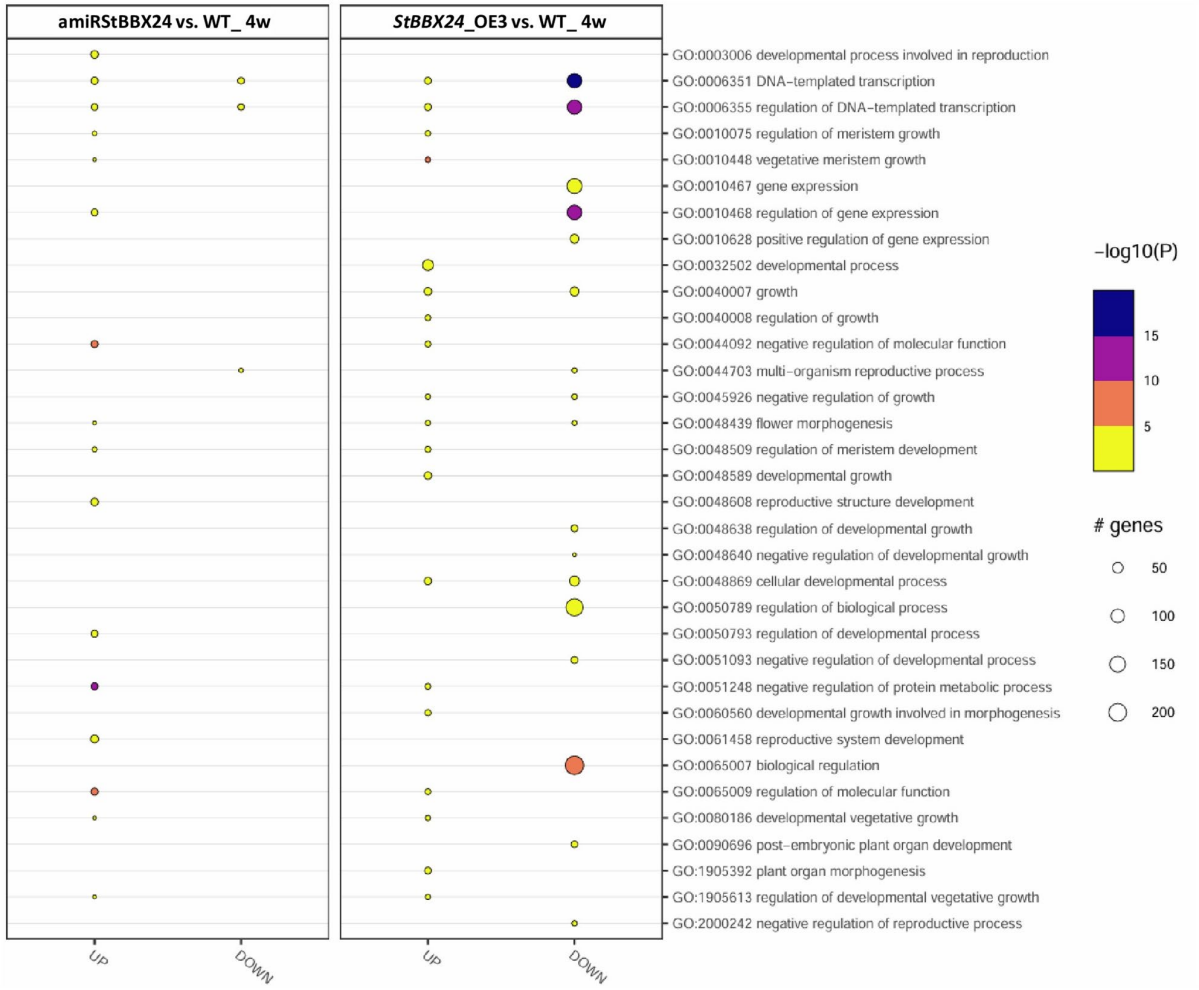
GO functional enrichment of differentially expressed genes in 4-week-old stolons of *StBBX24*-silenced and -overexpressed lines. The sizes of the wheels indicate the number of genes enriched in the GO term. The color of the wheels represents the Q-value.

The most represented biological processes, in which up- and down-regulated genes participate, concern regulation of transcription, development and cellular metabolism regulation, biosynthetic and metabolic processes, and reproductive system development (Fig.11).

**Fig. 12:**
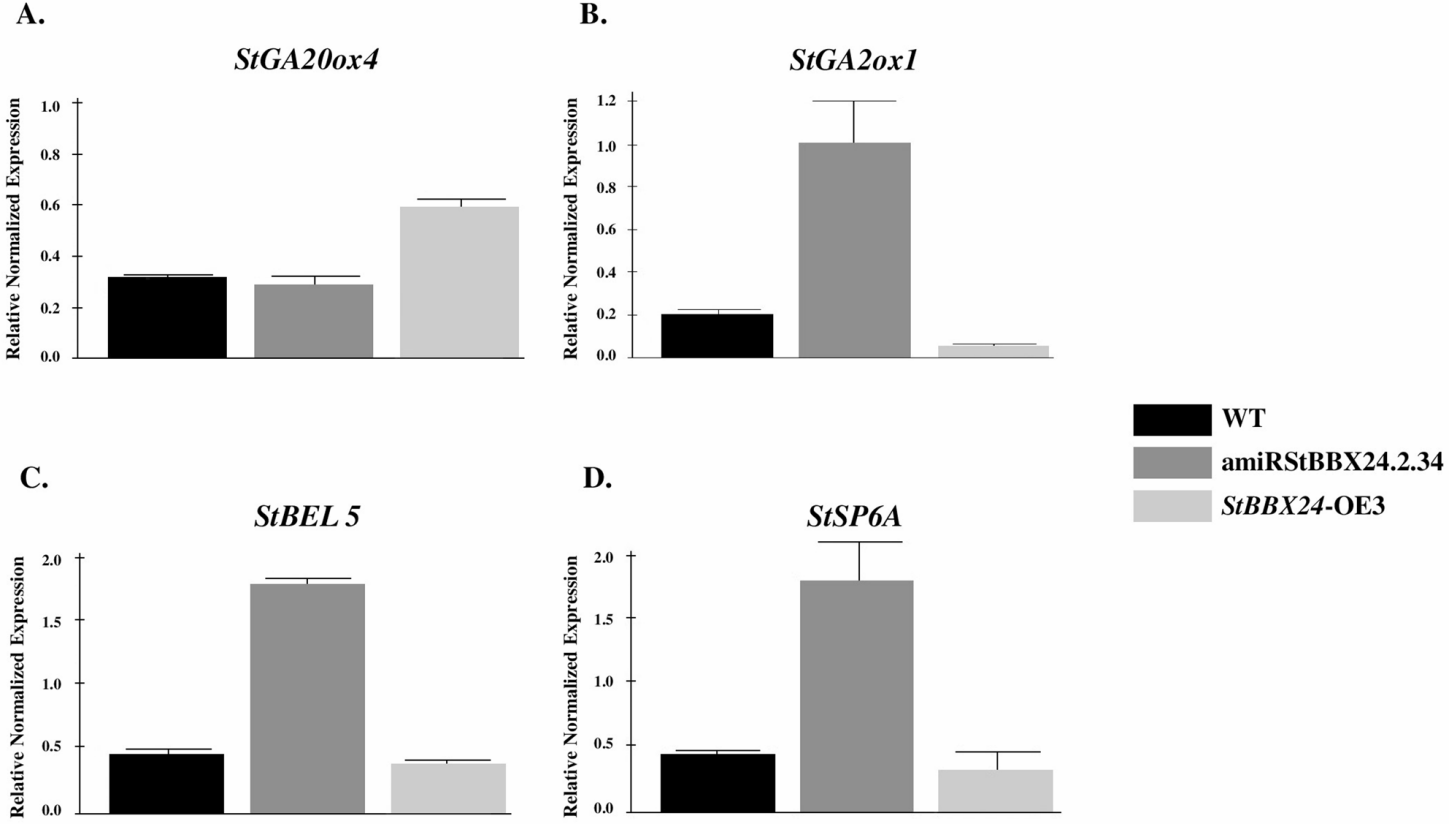
Expression profiles of tuber-related genes: *StGA20ox4* (**A**) (ID: PGSC0003DMG400000011), *StGA2ox1* (**B**) (ID: PGSC0003DMG400021095), *StBEL5* (**C**) (ID: PGSC0003DMG400019635) and *StSP6A *(**D**) (ID: PGSC0003DMG400023365) in 4-week-old *S. tuberosum StBBX24*-silenced (amiRStBBX24.2.34) and -overexpressed (*StBBX24-*OE3) lines, and WT plants at the initial stage of tuber formation using quantitative RT-PCR. RT-qPCR analyses were normalized using the threshold cycle (C1) values corresponding to the *StEF-1-α *reference gene. The normalized expression of the target gene (ΔΔCq) was calculated as the relative quantity of the target normalized to the quantities of the reference gene. The values presented are the means of two technical replicates from four independent biological samples.

Regarding the tuberization process, we examined the genes involved in regulating of tuber formation more closely. Most interestingly, one of the down-regulated genes in the *StBBX24*-OE3 line was *StBEL5* (PGSC0003DMG400019635, detected as differentially expressed by all 3 methods), a main activator of *StSP6A*, and the others were *StPOTH1* (PGSC0003DMG400013493, detected by StringTie) and *StGA2ox1* (PGSC0003DMG400002068, detected by StringTie). It was demonstrated that the StBEL5 interacts with StPOTH1 and, in complex, activates the expression of tuber formation-related genes, such as *StSP6A* and *StGA2ox1*. It is worth mentioning that the transcript level of the other two genes belonging to the BEL transcription factor family, i.e., *StBEL1-related homeotic protein 5* (PGSC0003DMG400005930) and *StBEL14* (PGSC0003DMG400012329), were also down-regulated in the *StBBX24*-OE3 line. In contrast, we noticed that the expression of *StGA20ox4* (*Gibberellin 20-oxidase 4*; PGSC0003DMG400000011) a repressor of tuber formation under tuber-inducing conditions, was raised in line overexpressing *StBBX24* (detected by all three methods, with StringTie being most sensitive). Meanwhile, in the *StBBX24*-silenced line, we observed elevated expression of *StGA2ox1* (*Gibberellin 2-oxidase 1*; PGSC0003DMG400021095), *StCOL3/StBBX3* (PGSC0003DMG400027475) and *StSuSy* (*SUCROSE SYNTHASE*; PGSC0003DMG400002895) (detected by all three methods, with StringTie being most sensitive). Then, we analyze changes in the transcript level of genes involved in tuberisation including *StBEL5*, *StGA2ox1*, *StSP6A* and *StGA20ox4* using RT-qPCR (Fig. 12A-D). We revealed that in the amiRBBX24.2.34 silenced line, *StBEL5*, *StSP6A* and *StGA2ox2* transcript levels were up-regulated as compared with WT (Fig. 12B-D), while *StGA20ox4* was slightly decreased (Fig. 12A). Changes in transcript abundance were also noticed in *StBBX24*-OE3 line compared with non-transformed plants. We observed that the transcript of *StGA2ox2*, *StSP6A* and *StSP3D* genes were down-regulated (Fig. 12B-D), while the expression of *StGA20ox4* was substantially increased (Fig. 12A).

In conclusion, the RNA-seq and RT-qPCR analysis revealed that the StBBX24 protein acts as a negative regulator of the transcription of genes essential in controlling floral induction and flower development as well as genes encoding key tuberization inducers at the early stage of tuber formation.

## Discussion

In this work, we set out to address the following questions: How does the clock-dependent StBBX24 protein, belonging to the B-box family in potato, suppress flowering and affect the efficiency of the tuberization process? Are the genes involved in the regulation of floral transition via StBBX24 circadian phase-specific? To identify the direct light/dark cycle targets regulated by StBBX24 and potentially involved in the flowering process of potato, we mapped the genome-wide binding profile of StBBX24 by performing chromatin immunoprecipitation coupled with high-throughput sequencing. The analysis of genome-wide direct binding and global gene expression changes revealed that the StBBX24 protein impacts flowering by regulating the expression of numerous genes, including those essential in flowering time and floral organ identity. Furthermore, transcriptome analysis of stolons at the hook stage of tuber formation confirmed the role of StBBX24 in the tuberization process. RNA-seq analyses of *StBBX24*-silenced and -overexpressed lines revealed that StBBX24 participates in the reproductive development of potato very likely by controlling the expression of genes critical in this process.

### StBBX24 mediates floral development via the control of the expression of flowering-related genes

As an internal biological timekeeper, the circadian clock in plants plays a crucial role in measuring day length and regulating flowering time on a 24-hour schedule. Circadian clock regulation requires a complex network of interactions between the biological clock components and numerous genes involved in floral development. We previously reported that the potato *StBBX24*-silenced lines revealed accelerated flowering, as compared with wild-type plants, while plants overexpressing *StBBX24* mostly did not flower at all [41]. These data unveil the role of StBBX24 protein as a negative regulator of floral induction. Moreover, we revealed that StBBX24 is predominantly present in the chromatin fraction which supports its substantial role in regulating gene expression at the transcript level [41]. Although the StBBX24 protein does not contain a well-characterized DNA-binding domain in its structure, its involvement in the regulation of the transcription of target genes was confirmed [85]. Here, to better understand how the StBBX24 protein mediates negative regulation of the vegetative to reproductive transition we aimed to identify its direct target genes involved in flowering, under both light and dark conditions. Interestingly, a substantial proportion of sites were found in intergenic regions, which is consistent with the fact that crucial regulatory elements, such as distal enhancers [86], can reside outside of immediate promoter regions. Among light-specific genes, one encoding CONSTANS interacting protein 2a was identified, an orthologue of the AtHAP5 (HEME ACTIVATOR PROTEIN 5) in Arabidopsis, which is a part of the CCAAT box binding trimeric complex AtHAP2/AtHAP3/AtHAP5. It was noticed that AtHAP3 and AtHAP5 subunits interact with CONSTANS (CO/BBX1) and the other CO-related protein, called AtCOL15, thus mediating the impact of CO on flowering time [87]. Similarly, Ben-Naim et al. [88] revealed the interaction between CO homolog and THAP5 protein in tomato (*Solanum lycopersicum*). They noticed that transgenic 35 S:*THAP5* Arabidopsis plants displayed the pronounced flowering-time effect under warmer long-day conditions, while at an optimal temperature, only two 35 S:*THAP5* transgenic lines showed a slight delay in flowering. Other putative targets of StBBX24, regardless of the phase of the circadian cycle, were *StRAPTOR1B* and *StSUP* genes. In Arabidopsis, the RAPTOR1B protein functions as a component of the nutrient-sensing TOR complex (TORC). It promotes flowering under long-day (LD) due to its impact on components of the photoperiod-dependent flowering regulatory pathway [83] while the *SUP* gene encodes a repressor of floral homeotic genes influencing floral development [89, 90]. We also paid attention to the light-specific StBBX24 binding peaks located ~ 8.6 kb of TSS related to flowering, i.e., *StSPL3*, *StSOC1* and *StFPF1* for which the role of StBBX24 protein as a distal transcriptional regulator cannot be excluded. Yamaguchi et al. [91] demonstrated that *SPL3* controls the expression of MADS-box genes: *StFUL* (*FRUITFULL*) and *StSOC1*, which have redundant roles in promoting flowering [92], independently of the FT/FD complex. In addition, it was also noted that the miR156-SPL3 module fulfils a crucial role in regulating ambient temperature-responsive flowering time in Arabidopsis via inducing changes in *FT* gene expression [93]. Another light-dependent target encodes the FPF1, which acts as a mobile signal accelerating early flowering in parallel with FT and induces the expression of the *SEPALLATA3* (*SEP3*) gene [94].

Interestingly, within all identified targets, we selected the most relevant motifs for the transcription factors regulating the expression of genes specific to light and dark conditions. We found that the ‘AY/HCYACGGD/RCCGTR’, the light- and dark-characteristic motif, is recognized by, i.e., the TCP transcription factor belonging to bHLH family and RAP2-4 protein from AP2-ERF family. It was revealed that numerous TCP proteins are involved in different processes related to plant reproductive development, including regulation of flowering time and development of flower organs [95]. In Arabidopsis, some members of TCP family, such as AtTCP7, AtTCP8, AtTCP14, and AtTCP15, function as positive regulators of flowering, whereas others, AtTCP18, AtTCP20, AtTCP22 and AtTCP23 act as flowering repressors [96–100]. Moreover, other representatives, i.e., AtTCP2, AtTCP3, AtTCP4, AtTCP10, and AtTCP24, participate in the switch from vegetative growth to reproductive development by direct induction of the key photoperiod-responsive regulator *CO*, an inducer of *FT* transcription [95, 101]. Similarly, the RAP2.4 transcription factor also plays a role in regulating the flowering time, likely by interacting with light and ethylene signaling pathways. Lin et al. [102] demonstrated that overexpression of *RAP2.4* in Arabidopsis led to delayed flowering, while loss-of-function mutants did not display any relevant phenotype. Unfortunately, none of the identified motifs were found in the promoter regions of StBBX24-targets related to flowering time and floral development, including *CONSTANS interacting protein 2a*, *StRAPTOR1B*, *StSUP*, *StSPL3*, *StSOC1* and *StFPF1* suggesting that the regulation of their expression by StBBX24 occurs very likely through binding to other regulatory elements.

Next, based on the observed phenotype, we paid more attention to genes associated with the flowering using *StBBX24*-silenced and -overexpressed lines at two different stages of shoot apex development. RNA-seq and RT-qPCR analyses revealed that the expression of several genes of the flowering regulatory pathway was affected in transgenic plants. Indeed, in 5-week-old *StBBX24*-silenced line, we noticed decreased expression levels of *StCOL3/StBBX3* and a gene *StSP5G* encoding flowering repressor, while the expression of the floral inducers *StSP6A* and *StSP3D* were up-regulated (Fig. 9). In potato, three tandemly arranged orthologs (*StCOL1*-*StCOL3*) of the CO protein were located on chr02 [7]. So far, it has been determined that the StCOL1 protein suppresses tuber formation under LD conditions, while the function of its two homologs in potato developmental processes has not yet been elucidated [7]. Furthermore, in Arabidopsis, a facultative LD plant, CO is a key inducer of the photoperiodic flowering response via regulation of *FT* gene transcription under LD. Conversely, Luccioni et al. [103] noticed that CO inhibits *FT* induction and delays flowering under SD conditions. Then we revealed that 5-week-old plants overexpressing *StBBX24* showed decreased transcript levels of some genes encoding floral homeotic proteins, including AGAMOUS, SEP1 (SEPALLATA 1) and FBP1, which play a crucial role in determining the identity of floral organs. It is worth mentioning that in addition to regulating flowering time, B-box proteins may also be implicated in flower development. The involvement of BBX19 and BBX24 proteins in the regulation of miRNA-lncRNA-TF module and trigger of floral organ development *Liriodendron chinense* was reported [104]. In addition, in *Oryza sativa*, two BBX proteins, OsBBX11 and OsBBX19, regulate the expression of *OsBTB97* participating in the development of rice spikelets [105]. On the other hand, in the 7-week-old *StBBX24*-silenced line, we observed significantly decreased transcript level of the *StBEL34*, *LSH1*, *KNOX10* and *StSPL9*, while the expression of *Floral homeotic protein AGAMOUS* and MADS-box transcription factor *FBP4*, were up-regulated. Interestingly, in line with *StBBX24* overexpression, the transcript abundance for *StMYB84*, an ortholog of the *AtMYB62*, was up-regulated. It was demonstrated that Arabidopsis plants overexpressing *MYB62* displayed suppressed expression of *SOC1* and *SUPERMAN*, molecular regulators of flowering [106]. The lack of significant changes in the expression of genes encoding key inducers and repressors of flowering in the *StBBX24*-silenced line in transcriptome analysis may be related to the observed transient silencing effect. While expression of *StBBX24* was confirmed to be down-regulated upon transformation in the silenced genotype, transcriptomics suggests this effect wanes over time. This is an important caveat for our results, which merits further investigation under other systems for altered gene expression. Notably, the overexpressing genotype registers with significant upregulation of *StBBX24* according to all three quantification methods, confirming the long-term expression changes in that genotype.

These data conclude that StBBX24 acts as a negative regulator of genes promoting floral induction and flower formation and as a positive regulator of those repressing these processes through a time-keeping mechanism related to day length.

### StBBX24 affects the tuberization process in potato

Our previous data showed overall increased tuber yields in *S. tuberosum StBBX24*-silenced lines as compared with WT plants and the lines with *StBBX24* overexpression [41]. Considering that there are several signalling pathways common to both flowering and tuberization, we analysed global transcriptomics at the initial stage of tuber formation using *StBBX24*-silenced and -overexpressed plants in the present work. Indeed, in the *StBBX24*-OE3 line, we observed significantly reduced expression of genes promoting tuberization, i.e., *StBEL5*,* StPOTH1* and *StGA2ox1* and elevated transcript level of genes encoding tuber formation repressor, i.e., *StGA20ox4*. Meanwhile, in the *StBBX24*-silenced line, the expression of genes promoting tuberization, i.e., *StGA2ox1* and *StSuSy* were up-regulated, in full agreement with the tuberization phenotype observed. In potato, the StBEL5 transcription factor fulfils a key role in tuber formation. It acts as a mobile signal, moving from the leaves through the phloem to stolon tips, and initiates the tuberization process. Banerjee et al. [107] indicated that overexpression of *StBEL5* causes earlier and increased tuber production, while inhibition of its expression reduces tuber yield. At the initial phase of tuberization, StBEL5 interacts with StPOTH1 (potato homeobox1) protein and in the complex they induce the expression of *StSP6A* and trigger the tuberization signal in leaves. It explains the markedly increased transcript level of *StSP6A* in the apical shoots of the 5-week-old *StBBX24*-silenced line. Amplification of signal takes place in stolons, where StBEL5 with StPOTH1 partner induces the transcription of both *StSP6A* and *StBEL5* [108–110]. Moreover, the StBEL5-StPOTH1 heterodimer also induces the expression of *StGA2ox1*, a critical metabolic gene of GAs, while inhibiting the expression of *StGA20ox1* gene encoding GAs biosynthetic enzymes to reduce the GA levels required for the tuberization onset.

These data indicate that StBBX24 protein is involved in potato storage organ formation as a repressor of genes promoting tuberization and as an inducer of those repressing this process, thus contributing to optimizing tuber yield.

## Conclusion

Our results characterized the StBBX24 protein as an essential component in the network controlling reproductive development. These studies indicate a high level of complexity in the interactions of the StBBX24 protein and its target genes depending on cycles of light and dark. One essential aim for future research will be to validate and functionally characterise the candidate genes and investigate their role in flowering time and tuber formation of cultivated potato. In other aspects, the isolation of protein partners interacting with StBBX24 will enable a better understanding of the role of StBBX24 in the regulatory network underlying reproductive system development, and provide valuable insights into the mode of action of BBX proteins.

## Supplementary Information

Supplementary Material 1.

Supplementary Material 2.

Supplementary Material 3.

Supplementary Material 4.

Supplementary Material 5.

Supplementary Material 6.

Supplementary Material 7.

Supplementary Material 8.

Supplementary Material 9.

Supplementary Material 10.

## Data Availability

All data generated and analyzed in this study are included in the published article and its supplementary information files. Additionally, RNA-seq raw data are available in the ArrayExpress repository (https://www.ebi.ac.uk/biostudies/arrayexpress/studies/E-MTAB-15322) under accession number E-MTAB-15322, while CHIP-seq raw data are available in the NCBI/SRA (Sequence Read Archive) (https://www.ncbi.nlm.nih.gov/sra/) under accession number PRJNA1284286.
