# Supplementary material for: CHIP-seq and transcriptomics reveal a new role of circadian-regulated StBBX24 protein in potato reproduction
Source: BMC Plant Biol. 2025 Dec 2;25:1702. doi: 10.1186/s12870-025-07811-0 (PMC12701598; doi:10.1186/s12870-025-07811-0)
Supplement: Supplementary file 9 — Supplementary Material 9. [file 12870_2025_7811_MOESM9_ESM.doc]

**Additional Figure**

**Figure S1.**


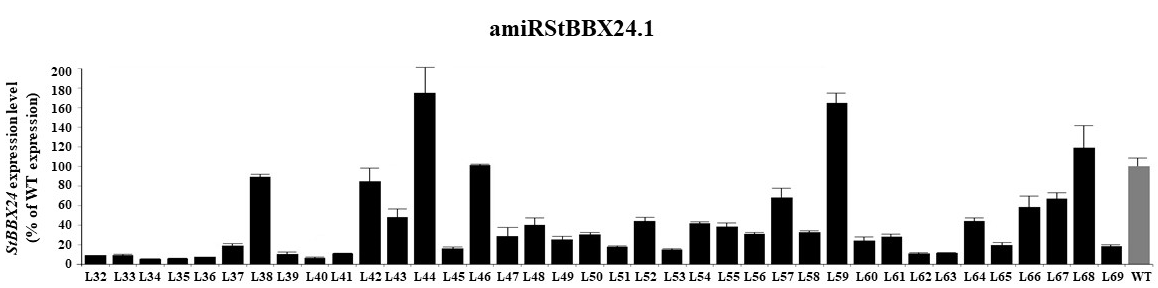
**(A)**

**
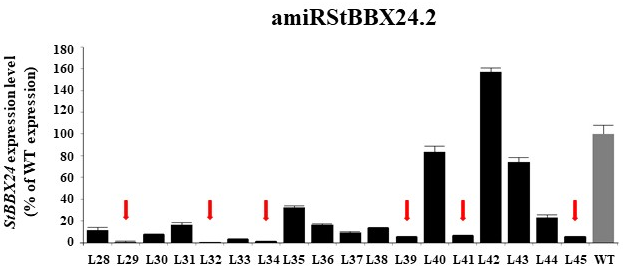
**

**
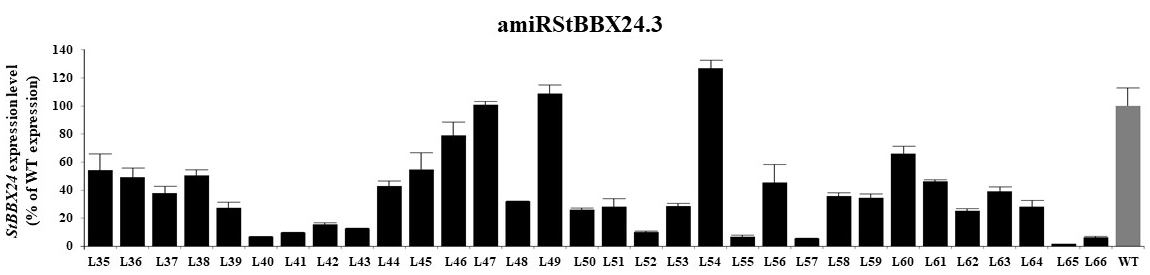
**

**(B)**

**StBBX24**


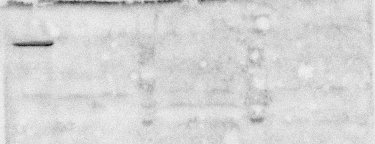


**WT L29 L32 L34 L39 L41 L45**

**amiRStBBX24.2**

**26 kDa**

**WT L29 L32 L34 L39 L41 L45**

**amiRStBBX24.2**


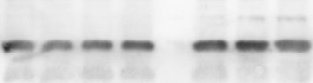


**17 kDa**

**H3**

**Figure S1.** Artificial miRNAs silence the expression of the *StBBX24* gene in *S. tuberosum* cv. Desirée at transcript and protein level. **(A)** Analysis of *StBBX24* transcript accumulation in amiRStBBX24 *S. tuberosum* transgenic lines by RT-qPCR. The arrows indicate transgenic lines showing significantly lower transcript level, comparing to WT control, selected to analysis of StBBX24 protein level by Western blot. **(B)** Western blot analysis of the presence of StBBX24 protein. in Desirée plants (control) and the selected transgenic lines (amiRStBBX24.2.29, amiRStBBX24.2.32, amiRStBBX24.2.34, amiRStBBX24.2.39, amiRStBBX24.2.41, amiRStBBX24.2.45). Histone H3 protein was used as an internal control in Western blot analysis. L – individual transgenic lines; WT – wild type.

**Figure S2.**

**
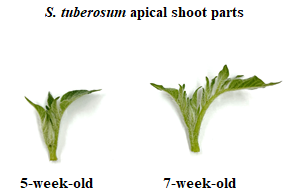
**

**Figure S2.** 5- and 7-week-old apical shoots of *Solanum tuberosum*, cv. Desirée.

**Figure S3.**

**
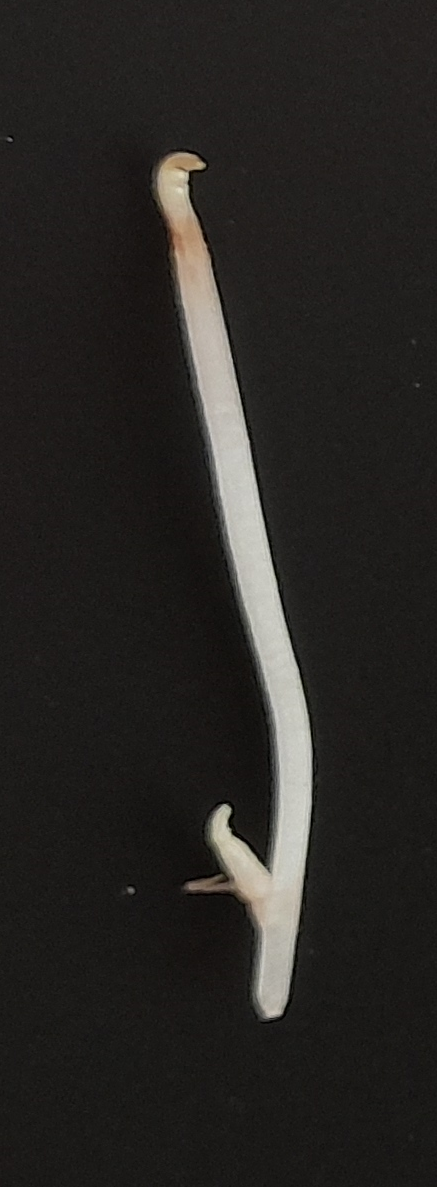
**

**Figure S3.** 4-week-old stolon of *Solanum tuberosum*, cv. Desirée at the hook stage.
