# Supplementary material for: CHIP-seq and transcriptomics reveal a new role of circadian-regulated StBBX24 protein in potato reproduction
Source: BMC Plant Biol. 2025 Dec 2;25:1702. doi: 10.1186/s12870-025-07811-0 (PMC12701598; doi:10.1186/s12870-025-07811-0)
Supplement: Supplementary file 10 — Supplementary Material 10. [file 12870_2025_7811_MOESM10_ESM.doc]

**Additional Tables**

**Table S1.** The primers used.

| **Primers used for construct preparation for *StBBX24* overexpression and chromatin immunoprecipitation** | |
| --- | --- |
| **StBBX24_C-tag_Fd** | 5′ -CACCATGAAGATCCAGTGTGATGTGT- 3’ |
| **StBBX24_C-tag_Rev** | 5′ -ACCAAGATCTGGGACAGTAAAATA- 3’ |
| **StBBX24_N-tag_Rev** | 5′ -TCAACCAAGATCTGGGACAGTAAA- 3’ |
| **Primers used for cDNA fragments amplification of the *StBBX24* gene in real-time PCR reactions** | |
| **StBBX24_qPCR_Fd** | 5’ -CAAGCTGCAATAAGGAAG- 3’ |
| **StBBX24_qPCR_Rev** | 5’ -CTCGACTCATAATCTGGAA- 3’ |
| **StEF1α_qPCR_Fd** | 5’ -CTGGTATGGTTAAGATGATTC- 3’ |
| **StEF1α_qPCR_Rev** | 5’ -GTCCTTCTTGTCAACATTC- 3’ |
| **StSP6A_qPCR_Fd** | 5’ –CCAGGTTGGCGTCAAAAT- 3’ |
| **StSP6A_qPCR_Rev** | 5’ –TGCGCGACGTCCTCCAGT- 3’ |
| **StSP3D_qPCR_Fd** | 5’ –TGGTTATGGTGGACCCAGAT- 3’ |
| **StSP3D_qPCR_Rev** | 5’ –GCCATCCTGGAGCATACACT- 3’ |
| **StSP5G_qPCR_Fd** | 5’ –CCAAACCCTAGCAATCCAAA- 3’ |
| **StSP5G_qPCR_Rev** | 5’ –TTGCTGGAACAACACGAAAA- 3’ |
| **StBEL5_qPCR_Fd** | 5’ –ATAGTACCATCGTCGACG- 3’ |
| **StBEL5_qPCR_Rev** | 5’ –ACGCCAAGTCCGTGGTGA- 3’ |
| **StGA2ox1_qPCR_Fd** | 5’ –CAACAACAGTGTCTGAATTAGC- 3’ |
| **StGA2ox1_qPCR_Rev** | 5’ –TCGATTCATTCGAACGTAACAA- 3’ |
| **StGA20ox4_qPCR_Fd** | 5’ –AGGGACAGGACCTCATTGTG- 3’ |
| **StGA20ox4_qPCR_Rev** | 5’ –TGCTCTGTGCAAGCAACTCT- 3’ |
| **StCOint_qPCR_Fd** | 5’ –CAAGGCATGTGAGCTTTTCA- 3’ |
| **StCOint_qPCR_Rev** | 5’ –CCTCGGAACAATGTCAACAA- 3’ |
| **StSPL_qPCR_Fd** | 5’ –TTCTTCGGAGGATTCACCAC- 3’ |
| **StSPL_qPCR_Rev** | 5’ –AATCCCTGCCATCTCCTCTT- 3’ |
| **StRAPTOR1B_qPCR_Fd** | 5’ –TCTGAGCCCCAACCTGAGGT- 3’ |
| **StRAPTOR1B_qPCR_Rev** | 5’ –ACTCGAACCAAAGGGCTTCC- 3’ |
| **StMADS47_qPCR_Fd** | 5’ –AACGTGGTGAACCGCTACAT- 3’ |
| **StMADS47_qPCR_Rev** | 5’ –ATTGTGCGATCCAACTCCTC- 3’ |
| **StSUP_qPCR_Fd** | 5’ –GAGGAAGATACTGGCAATTC- 3’ |
| **StSUP_qPCR_Rev** | 5’ –CACTACGGTGCACATTCATG- 3’ |

**Table S2.** Sequencing throughput and mapping results obtained for CHIP and input samples.

| **Statistics filtering** | **CHIP_BBX24_L1** | **CHIP_BBX24_L2** | **InPut_L** | **CHIP_BBX24_D1** | **CHIP_BBX24_D2** | **InPut_D** |
| --- | --- | --- | --- | --- | --- | --- |
| Both Surviving Reads | 27563076 | 26802997 | 27479086 | 28490880 | 23756403 | 29312194 |
| Both Surviving Read Percent | 97.93% | 97.86% | 98.11% | 97.7% | 97.95% | 98.34% |
| Forward Only Surviving Reads | 281638 | 287431 | 238598 | 314240 | 246829 | 199981 |
| Forward Only Surviving Read Percent | 1% | 1.05% | 0.85% | 1.08% | 1.02% | 0.67% |
| Reverse Only Surviving Reads | 208768 | 205606 | 211927 | 245635 | 176223 | 237348 |
| Reverse Only Surviving Read Percent | 0.74% | 0.75% | 0.76% | 0.84% | 0.73% | 0.8% |
| Dropped eads | 93031 | 92247 | 79440 | 110338 | 73385 | 58343 |
| Dropped Read Percent | 0.33% | 0.34% | 0.28% | 0.38% | 0.3% | 0.2% |

**Table S3.** Mapping statistics of merged files.

| **Samples** | **Quality Control Passed Reads** | **Secondary Reads** | **Mapped Reads** | **Percent of Mapped Reads** | **Paired Reads** | **Properly Paired Reads** | **Percent of Properly Paired Reads** |
| --- | --- | --- | --- | --- | --- | --- | --- |
| **CHIP_BBX24_L** | 105771800 | 4728259 | 105395337 | 99.64% | 101043541 | 93684006 | 92.72% |
| **CHIP_BBX24_D** | 102633304 | 5763396 | 102254115 | 99.63% | 96869908 | 88781298 | 91.65% |

**Table S4.** Transcriptional regulators targeted by StBBX24 under light and dark conditions.

| **LIGHT** | | **DARK** | |
| --- | --- | --- | --- |
| **TRANSCRIPTION FACTORS (TF)** | | | |
| **Gene ID** | **Family** | **Gene ID** | **Family** |
| PGSC0003DMG400035501 | MADS-domain | PGSC0003DMG400006209 | MYB-HB-like |
| PGSC0003DMG400029162 | C3HC2HC or C6HC | PGSC0003DMG400008761 | MYB-HB-like |
| PGSC0003DMG400021310 | C2H2 | PGSC0003DMG400018060 | C2H2 |
| PGSC0003DMG400006209 | MYB-HB-like | PGSC0003DMG400004205 | C2H2 |
| PGSC0003DMG400033904 | MYB-HB-like | PGSC0003DMG400045009 | bHLH |
| PGSC0003DMG400005527 | SSXT | PGSC0003DMG400035432 | MYB-HB-like |
| PGSC0003DMG400013966 | MYB-HB-like | PGSC0003DMG400015104 | WRKY |
| PGSC0003DMG400007174 | MYB-HB-like | PGSC0003DMG400004499 | Homobox-WOX |
| PGSC0003DMG400034516 | MYB-HB-like | PGSC0003DMG400013105 | C2C2-Dof |
| PGSC0003DMG400037815 | MYB-HB-like | PGSC0003DMG400013213 | HD-ZIP |
| PGSC0003DMG400017813 | AP2-EREBP | PGSC0003DMG400031777 | Homobox-WOX |
| PGSC0003DMG400008649 | AS2-LOB | PGSC0003DMG400039628 | AP2-EREBP |
| PGSC0003DMG400000441 | C2C2-Dof | PGSC0003DMG400012599 | C3H |
| PGSC0003DMG400034428 | HSF-type-DNA-binding | PGSC0003DMG400026328 | WD40-like |
| PGSC0003DMG400026332 | NAM | PGSC0003DMG400033694 | HD-ZIP |
| PGSC0003DMG400015565 | Hap3/NF-YB | PGSC0003DMG400025355 | ssDNA-binding-TF |
| PGSC0003DMG400025355 | ssDNA-binding-TF | PGSC0003DMG400003357 | Hap3/NF-YB |
| PGSC0003DMG400003357 | Hap3/NF-YB | PGSC0003DMG400015828 | AP2-EREBP |
| PGSC0003DMG400015828 | AP2-EREBP | PGSC0003DMG400035501 | MADS-domain |
| PGSC0003DMG400028638 | MADS-type1 | PGSC0003DMG400033904 | MYB-HB-like |
| PGSC0003DMG400017761 | C2H2 | PGSC0003DMG400013966 | MYB-HB-like |
| PGSC0003DMG400017762 | C2H2 | PGSC0003DMG400037815 | MYB-HB-like |
| PGSC0003DMG400002337 | C2C2-GATA | PGSC0003DMG400006482 | AP2-EREBP |
| PGSC0003DMG400010912 | C2H2 | PGSC0003DMG400018986 | bHLH |
| PGSC0003DMG400008761 | MYB-HB-like | PGSC0003DMG400037529 | B3-Domain |
| PGSC0003DMG400016363 | TCP | PGSC0003DMG400029510 | C2H2 |
| PGSC0003DMG400018060 | C2H2 | PGSC0003DMG400004515 | AP2-EREBP |
| PGSC0003DMG400014990 | C2H2 | PGSC0003DMG400036493 | AP2-EREBP |
| PGSC0003DMG400024024 | MADS-type1 | PGSC0003DMG400039175 | WRKY |
| PGSC0003DMG400038949 | AP2-EREBP | PGSC0003DMG400026332 | NAM |
| PGSC0003DMG400004205 | C2H2 | PGSC0003DMG400028638 | MADS-type1 |
| PGSC0003DMG400022689 | MYB-HB-like | PGSC0003DMG400021778 | C2H2 |
| PGSC0003DMG400045009 | bHLH | PGSC0003DMG400040417 | HD-ZIP |
| PGSC0003DMG400035432 | MYB-HB-like | PGSC0003DMG400016896 | NAM |
| PGSC0003DMG400011316 | MADS-type1 | PGSC0003DMG400018834 | WD40-like |
| PGSC0003DMG401006284 | SBP | PGSC0003DMG400006988 | MYB-HB-like |
| PGSC0003DMG400000120 | C2C2-Dof | PGSC0003DMG400045703 | B3-Domain |
| PGSC0003DMG400029773 | Lambda-DB | PGSC0003DMG400003404 | C2H2 |
| PGSC0003DMG400015104 | WRKY | PGSC0003DMG402008471 | WD40-like |
| PGSC0003DMG400046652 | MADS-type1 | PGSC0003DMG400021599 | MYB-HB-like |
| PGSC0003DMG400038730 | MADS-type1 | PGSC0003DMG400030055 | C2H2 |
| PGSC0003DMG400037967 | NAM | PGSC0003DMG400012099 | WD40-like |
| PGSC0003DMG400015498 | C2H2 | PGSC0003DMG400019600 | C2H2 |
| PGSC0003DMG400004499 | Homobox-WOX | PGSC0003DMG400001161 | bHLH |
| PGSC0003DMG400013105 | C2C2-Dof | PGSC0003DMG400026644 | MYB-HB-like |
| PGSC0003DMG400013213 | HD-ZIP | PGSC0003DMG400017035 | WD40-like |
| PGSC0003DMG400010194 | MYB-HB-like | PGSC0003DMG400037372 | MADS-type1 |
| PGSC0003DMG400031777 | Homobox-WOX | PGSC0003DMG400001749 | MYB-HB-like |
| PGSC0003DMG400039628 | AP2-EREBP | PGSC0003DMG400002098 | C2H2 |
| PGSC0003DMG400012653 | AS2-LOB | PGSC0003DMG400038007 | C2H2 |
| PGSC0003DMG400012599 | C3H | PGSC0003DMG400039299 | bHLH |
| PGSC0003DMG400026328 | WD40-like | PGSC0003DMG400025700 | WD40-like |
| PGSC0003DMG400030271 | C2H2 | PGSC0003DMG400024171 | C3H |
| PGSC0003DMG400008340 | MYB-HB-like | PGSC0003DMG400007690 | C2H2 |
| PGSC0003DMG401024252 | MADS-type1 | PGSC0003DMG400009276 | TCP |
| PGSC0003DMG400017776 | WOX | PGSC0003DMG400002185 | AP2-EREBP |
| PGSC0003DMG400033694 | HD-ZIP | PGSC0003DMG400013589 | MADS-type1 |
| PGSC0003DMG400036493 | AP2-EREBP | PGSC0003DMG400025559 | WD40-like |
| PGSC0003DMG400039175 | WRKY | PGSC0003DMG400045649 | MADS-type1 |
| PGSC0003DMG400039636 | Hap3/NF-YB | PGSC0003DMG400008774 | C2H2 |
| PGSC0003DMG402015259 | Hap3/NF-YB | PGSC0003DMG400016753 | Hap3/NF-YB |
| PGSC0003DMG400013184 | C2H2 | PGSC0003DMG400000278 | C3H |
| PGSC0003DMG400006482 | AP2-EREBP | PGSC0003DMG400043801 | AP2-EREBP |
| PGSC0003DMG400018986 | bHLH | PGSC0003DMG400015828 | AP2-EREBP |
| PGSC0003DMG400037529 | B3-Domain | PGSC0003DMG400026460 | Homobox-WOX |
| PGSC0003DMG400029510 | C2H2 | PGSC0003DMG400030389 | MYB-HB-like |
| PGSC0003DMG400004515 | AP2-EREBP | PGSC0003DMG400004371 | MYB-HB-like |
| PGSC0003DMG400039820 | bZIP | PGSC0003DMG400017759 | MADS-type1 |
| PGSC0003DMG400003459 | C2H2 | PGSC0003DMG400015058 | C2H2 |
| PGSC0003DMG400024102 | TUBBY | PGSC0003DMG400022695 | AP2-EREBP |
| PGSC0003DMG400023619 | AP2-EREBP | PGSC0003DMG400022651 | C3H |
| PGSC0003DMG400023618 | C2H2 | PGSC0003DMG400025976 | bHLH |
| PGSC0003DMG400041851 | C2H2 | PGSC0003DMG400000062 | MADS-type1 |
| PGSC0003DMG400034310 | SBP | PGSC0003DMG400015104 | WRKY |
| PGSC0003DMG400021778 | C2H2 | PGSC0003DMG400037967 | NAM |
| PGSC0003DMG400021777 | C2H2 | PGSC0003DMG400003561 | Homobox-WOX |
| PGSC0003DMG400040417 | HD-ZIP | PGSC0003DMG400012683 | GRAS |
| PGSC0003DMG400016896 | NAM | PGSC0003DMG400001386 | C2H2 |
| PGSC0003DMG400018834 | WD40-like | PGSC0003DMG400012825 | C2C2-Dof |
| PGSC0003DMG400038060 | MADS-type1 | PGSC0003DMG400040625 | MADS-type1 |
| PGSC0003DMG400001223 | NAM | PGSC0003DMG400040127 | MADS-type1 |
| PGSC0003DMG400007027 | AP2-EREBP | PGSC0003DMG400026736 | SBP |
| PGSC0003DMG400006988 | MYB-HB-like | PGSC0003DMG400020621 | EIL |
| PGSC0003DMG400016004 | AP2-EREBP | PGSC0003DMG400024853 | GRAS |
| PGSC0003DMG400045703 | B3-Domain | PGSC0003DMG400002135 | C2H2 |
| PGSC0003DMG400003404 | C2H2 | PGSC0003DMG400023582 | HD-ZIP |
| PGSC0003DMG402008471 | WD40-like | PGSC0003DMG400023619 | AP2-EREBP |
| PGSC0003DMG400021599 | MYB-HB-like | PGSC0003DMG400023618 | C2H2 |
| PGSC0003DMG400025279 | MADS-type1 | PGSC0003DMG400041851 | C2H2 |
| PGSC0003DMG400019600 | C2H2 | PGSC0003DMG400035405 | B3-Domain |
| PGSC0003DMG400002098 | C2H2 | PGSC0003DMG400038060 | MADS-type1 |
| PGSC0003DMG400026068 | C2H2 | PGSC0003DMG400018439 | MYB-HB-like |
| PGSC0003DMG400030055 | C2H2 | PGSC0003DMG400018427 | MYB-HB-like |
| PGSC0003DMG400012099 | WD40-like | PGSC0003DMG400035463 | MADS-type1 |
| PGSC0003DMG400029191 | C2C2-Dof | PGSC0003DMG400025505 | NAM |
| PGSC0003DMG400023248 | C2H2 | PGSC0003DMG400019017 | bHLH |
| PGSC0003DMG400020432 | WRKY | PGSC0003DMG400015659 | bHLH |
| PGSC0003DMG400038007 | C2H2 | PGSC0003DMG400031490 | Homobox-WOX |
| PGSC0003DMG400004470 | MYB | PGSC0003DMG400027599 | bZIP |
| PGSC0003DMG400029147 | MADS-type1 | PGSC0003DMG400027582 | WRKY |
| PGSC0003DMG400033616 | C2H2 | PGSC0003DMG400027959 | bHLH |
| PGSC0003DMG400001161 | bHLH | PGSC0003DMG400028896 | MYB-HB-like |
| PGSC0003DMG400036318 | B3-Domain | PGSC0003DMG400012099 | WD40-like |
| PGSC0003DMG400017509 | Znf-LSD | PGSC0003DMG400023295 | AP2-EREBP |
| PGSC0003DMG400026644 | MYB-HB-like | PGSC0003DMG400019092 | NAM |
| PGSC0003DMG400017035 | WD40-like | PGSC0003DMG400009773 | ARF |
| PGSC0003DMG400005560 | bHLH | PGSC0003DMG400008781 | TIFY |
| PGSC0003DMG400020807 | bHLH | PGSC0003DMG400044009 | AS2-LOB |
| PGSC0003DMG400023099 | WRKY | PGSC0003DMG400037980 | bZIP |
| PGSC0003DMG400037372 | MADS-type1 | PGSC0003DMG400026644 | MYB-HB-like |
| PGSC0003DMG400018950 | bHLH | PGSC0003DMG400016841 | WD40-like |
| PGSC0003DMG401001493 | WD40-like | PGSC0003DMG400046191 | WD40-like |
| PGSC0003DMG400001749 | MYB-HB-like |  |  |
| PGSC0003DMG400013159 | WD40-like |  |  |
| **CHROMATIN REGULATORS** | | | |
| **Gene ID** | **Family** | **Gene ID** | **Family** |
| PGSC0003DMG400012086 | - | PGSC0003DMG400012086 | - |
| PGSC0003DMG400036423 | - | PGSC0003DMG400036423 | - |
| PGSC0003DMG400044033 | - | PGSC0003DMG400044033 | - |
| PGSC0003DMG400034096 | SET | PGSC0003DMG400034096 | SET |
| PGSC0003DMG400038819 | PHD | PGSC0003DMG400038819 | PHD |
| PGSC0003DMG400010980 | SET | PGSC0003DMG400010980 | SET |
| PGSC0003DMG400011962 | PHD | PGSC0003DMG400024125 | Bromodomain |
| PGSC0003DMG400031304 | SET | PGSC0003DMG400031307 | SET |
| PGSC0003DMG400033921 | PHD | PGSC0003DMG400023442 | DDT |
| PGSC0003DMG400033659 | SNF2 |  |  |
| PGSC0003DMG400040275 | SNF2 |  |  |
| PGSC0003DMG400002152 | PHD |  |  |
| PGSC0003DMG400026134 | PHD |  |  |
| PGSC0003DMG400022994 | SET |  |  |
| PGSC0003DMG400005381 | PHD |  |  |
| PGSC0003DMG400043599 | PHD |  |  |
| **TRANSCRIPTION REGULATORS** | | | |
| **Gene ID** | | **Gene ID** | |
| PGSC0003DMG400038899 | | PGSC0003DMG400038899 | |
| PGSC0003DMG400041706 | | PGSC0003DMG400041706 | |
| PGSC0003DMG400007237 | | PGSC0003DMG400007237 | |
| PGSC0003DMG400007716 | | PGSC0003DMG400007716 | |
| PGSC0003DMG400000512 | | PGSC0003DMG400025731 | |
| PGSC0003DMG400021403 | | PGSC0003DMG400023206 | |
| PGSC0003DMG400031871 | | PGSC0003DMG400046664 | |
|  | | PGSC0003DMG400046044 | |
|  | | PGSC0003DMG400023442 | |

Genes common to light and darkness are highlighted.

| **Sample** | **Raw total reads** | **Raw total length (Gbp)** | **Post-trimming total reads** | **% pseudoaligned (kallisto)** | **% aligned (STAR)** |
| --- | --- | --- | --- | --- | --- |
| amiRStBBX24_AP_5_1 | 85874274 | 12,96701537 | 85810466 | 83,11116036 | 89,83997114 |
| amiRStBBX24_AP_5_2 | 61774082 | 9,327886382 | 61724810 | 83,98301105 | 90,52326561 |
| amiRStBBX24m_AP_7_1 | 76041454 | 11,48225955 | 75978700 | 82,56396069 | 88,62161961 |
| amiRStBBX24_AP_7_2 | 61109304 | 9,227504904 | 61079512 | 83,10266133 | 89,90513755 |
| amiRStBBX24_ST_4_1 | 63323958 | 9,561917658 | 63270518 | 81,62572337 | 88,3086406 |
| amiRStBBX24_ST_4_2 | 90136598 | 13,6106263 | 90057512 | 82,17887254 | 89,70794365 |
| StBBX24-OE3_AP_5_1 | 70940986 | 10,71208889 | 70870250 | 84,67951221 | 91,01966825 |
| StBBX24-OE3_AP_5_2 | 63718434 | 9,621483534 | 63664732 | 84,68883212 | 90,9542799 |
| StBBX24-OE3_AP_7_1 | 64926620 | 9,80391962 | 64861936 | 83,7436058 | 89,98421271 |
| StBBX24-OE3_AP_7_2 | 64699402 | 9,769609702 | 64631528 | 83,936852 | 89,95622664 |
| StBBX24-OE_ST_4_1 | 60435814 | 9,125807914 | 60365274 | 84,42996548 | 90,9020664 |
| StBBX24-OE_ST_4_2 | 64287908 | 9,707474108 | 64228376 | 84,36865351 | 92,09993642 |
| WT_AP_5_1 | 57065574 | 8,616901674 | 57037610 | 81,9537705 | 89,8247505 |
| WT_AP_5_2 | 67721610 | 10,22596311 | 67687682 | 82,91318352 | 89,46221994 |
| WT_AP_7_1 | 62402108 | 9,422718308 | 62332792 | 82,90031353 | 89,34133429 |
| WT_AP_7_2 | 52692402 | 7,956552702 | 52668268 | 82,35087586 | 90,28611702 |
| WT_ST_4_1 | 64839114 | 9,790706214 | 64763998 | 83,27806137 | 88,51323655 |
| WT_ST_4_2 | 68904148 | 10,40452635 | 68841544 | 82,90339624 | 89,54742624 |

**Table S5.** Quality statistics of raw sequencing data and analysis of the alignment of processed data to the reference genome sequence.
